# Concordant and discordant gene expression patterns in mouse strains identify best-fit animal model for human tuberculosis

**DOI:** 10.1038/s41598-017-11812-x

**Published:** 2017-09-21

**Authors:** Teresa Domaszewska, Lisa Scheuermann, Karin Hahnke, Hans Mollenkopf, Anca Dorhoi, Stefan H. E. Kaufmann, January Weiner

**Affiliations:** Max Planck Institute for Infection Biology, Department of Immunology, Charitéplatz 1, D-10117 Berlin, Germany

## Abstract

Immunity in infection, inflammation and malignancy differs markedly in man and mouse. Still, we learn about human immunity in large extent from experimental mouse models. We propose a novel data integration approach which identifies concordant and discordant gene expression patterns of the immune responses in heterologous data sets. We have conducted experiments to compare human and murine transcriptional responses to *Mycobacterium tuberculosis* (Mtb) infection in whole blood (WB) as well as macrophages and compared them with simulated as well as publicly available data. Our results indicate profound differences between patterns of gene expression in innate and adaptive immunity in man and mouse upon Mtb infection. We characterized differential expression of T-cell related genes corresponding to the differences in phenotype between tuberculosis (TB) highly and low susceptible mouse strains. Our approach is general and facilitates the choice of optimal animal model for studies of the human immune response to a particular disease.

## Introduction

The mouse has been the animal of choice for immunological studies for a century. It markedly broadened our knowledge of the structure and function of mammalian immune systems as well as of disease mechanisms. Despite high evolutionary pressure on immune systems^1^ and evolutionary distance between mouse and man^2^, the principles of the immune systems of these two species remain remarkably similar. Main discrepancies include the proportions of myeloid and lymphoid cells in blood, broader repertoire of B and T lymphocytes in human and heterogeneity in the repertoire of innate and adaptive immune signaling molecules^2,3^. High throughput genetic technologies have raised questions about the value of the murine system for modeling human diseases on the gene expression level^4,5^. However, comparison between heterologous data sets based on different technology platforms (as is the case for human and murine studies) presents a marked challenge, as the data cannot be aggregated and evaluated within a simple statistical framework such as linear modeling.

A study by Shay *et al*.^6^ provided a comparison of transcriptional profiles of seven non stimulated murine and human cell lineages collected during immune development and showed that the global expression profiles of corresponding cell types are similar between the two species. In contrast, in 2014 Lin *et al*.^7^ pointed to differences in human and murine transcriptomes. They described groups of genes that are tissue-specific or ubiquitous, and identified a subset of the latter which drives species-specific expression. In first approaches towards evaluating similarity of immune responses to specific stimuli, Seok *et al*.^4^ and Takao & Miyakawa^5^ employed the same data sets from total blood leukocytes from patients and corresponding mouse models to calculate correlations in murine and human gene expression. The outcome profoundly varied due to different biological and statistical assumptions of the two groups and resulted in contradictory verdicts about concordance of murine and human transcriptional responses. Most recently, a collection of over 5,000 immune system-specific gene sets based on publicly available data sets from mice and man was compiled^8^. This collection facilitates access to gene modules regulated concordantly in immunologically relevant comparisons of various cell-state perturbations and diseases from either human or murine studies. Such analysis can be followed by identification of genes which drive phenotypic differences in both species.

Yet, none of the published studies provide a universal solution to comparisons of transcriptome profiles from heterogeneous data sets. Notably, these approaches interpret lack of evidence for similarity as evidence of lack thereof, and a targeted approach aiming at detecting discordantly regulated elements of the immune response has not been attempted. The created gene collections apply exclusively to human and murine studies^8^ and the implemented solutions are based on correlation coefficients^4,5^. The drawback of such an approach is that a group of orthologous genes regulated in opposite manner (discordantly) upon infection can nonetheless show positive correlation (Figure S1). Reciprocally, if the definition of concordant gene expression is based on direction of regulation (up- or down-regulated) alone, the precision of the estimated changes in gene expression (in terms of confidence intervals, p-value or effect size) is not taken into account. This can result in lack of attribution of a major biological importance to genes playing crucial roles in a given disease.

Here, we introduce a method which allows both, identifying highly concordantly as well as highly discordantly regulated gene sets between two organisms. The method is based on measuring concordance using directionality of change weighted by the magnitude of gene expression change in two heterologous data sets (for example, human and murine) and associated precision of its estimate. To this end, the approach combines a novel measure of similarity with gene set enrichment (GSE) analysis. To validate our approach, we identified modules of genes concordantly and discordantly expressed in WB during TB in human populations from different regions and two different murine TB models. We then verified whether the differences found in WB are present also in human and murine macrophages.

Macrophages are pivotal for both innate and acquired immune responses to TB^9^. The number of macrophage precursors in blood of healthy human equals on average 200,000/ml of blood, i.e. 5–6% of the total white cell count^10^, and in mice around 60,000/ml of blood, constituting around 6% of circulating leukocytes^11^. Upon Mtb infection, the tissue resident macrophages engulf and constrain bacteria in the lung and recruit circulating monocytes and other leukocytes to the site of infection. This ultimately leads to formation of granulomas in humans and granuloma-like lesions in mice. Therefore, macrophages are one of the most important cell types involved in TB pathogenesis and protection.

Blood provides an easily accessible source of information about the state of an organism and WB samples remain the primary source of biomarkers of pathology, including infection^12^. WB cell transcriptome profiles are thought to illustrate a systemic immune response as blood contains cells and molecules of the immune system and is the carrier of metabolites between different tissues. WB cell composition in mouse and man is not directly comparable given that it varies in the ratio of neutrophils and lymphocytes (neutrophils comprise 50–70% of human and 10–25% of mouse WB cells, while lymphocytes comprise 30–50% of human and 75–90% of mouse WB cells^2^). However, states of infection drive changes in blood composition in both types of host, for example emergency granulopoesis and neutrophilia^13–15^.

Murine models of TB include the highly susceptible 129S2 strain and the low susceptible C57BL/6 strain^16^. Several genetic differences between the two strains, e.g. a mutation in Casp11 locus in 129S2 strain or mutation in Nramp1 in C57BL/6 mice, contribute to the difference in susceptibility phenotype of the two strains to infection with intracellular pathogens^17,18^. However, precise mechanisms of their differing resistance to TB have not been explained so far and it is speculated that they are also related to the occurrence and scale of inflammation, triggered by IFN production^19–21^. The 129S2 strain succumbs to the disease within 40 days post infection (p.i.) which is strongly related to exuberant interferon (IFN) type I signaling^19^, whereas the C57BL/6 strain controls the infection for an extended period of time (between 200 and 300 days)^16,22^. During disease progression, 129S2 mice develop necrotic lesions more similar to granulomas found in man, while C57BL/6 mice form smaller, organized lesions with necrosis evident only in the very late stage of infection^23^.

For these reasons we collected new data and acquired publicly available data from WB of TB patients from different geographic regions, from 129S2 and C57BL/6 mice infected with Mtb, as well as from healthy controls and compared the transcriptional responses to Mtb infection. These *in-vivo* data were complemented by *in-vitro* Mtb infection experiments with human and murine macrophages. Analysis of data revealed pronounced differences in gene expression upon Mtb infection in the highly and low susceptible mouse strain and identified concordant and discordant immune responses in TB between man and each of the two mouse strains which impacts on disease modeling.

## Results

### Definition of a novel similarity measure (disco.score)

**Figure 1:**
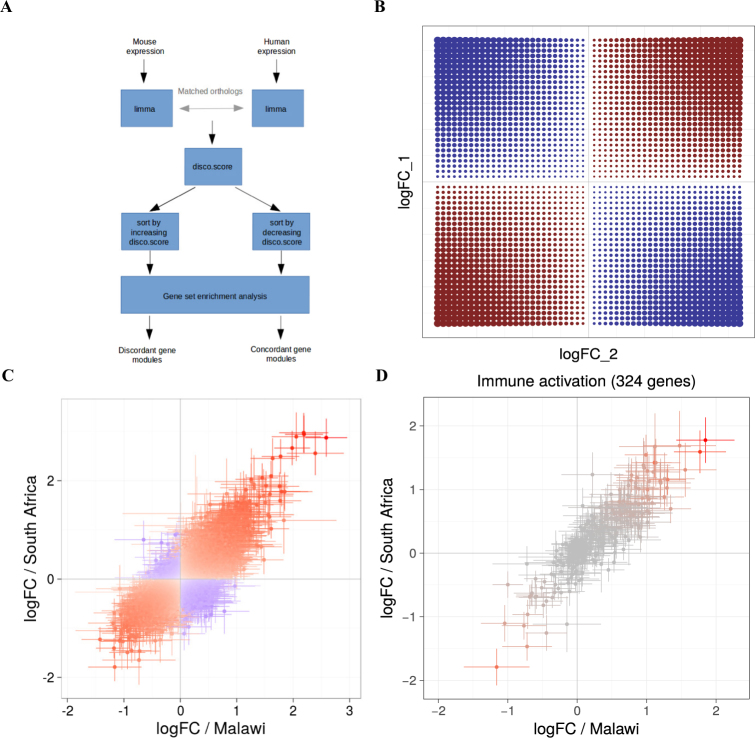
Concordance and discordance in gene expression data. (**A**) Algorithm used to identify concordant and discordant gene modules. The log fold changes and p-values between groups were calculated with R-package *limma*. The orthologous genes or genes corresponding to each other (if compared data sets derive from two groups of the same species) were mapped to each other. Then, disco.score was calculated for each pair of corresponding genes. GSE analysis was performed on the list of genes sorted by increasing disco.score to distinguish discordant gene modules and on the list of genes sorted by decreasing disco.score to distinguish concordant gene modules. (**B**) Theoretical distribution of disco.score function depending on log_2_FC values of both species. Increasing intensity of the red color indicates increase in disco.score and illustrates higher degree of similarity between human and murine gene expression. Increasing intensity of the blue color indicates decrease in negative disco.score and a higher degree of dissimilarity between human and murine gene expression. (**C**) Distribution of disco.score in the assessment of similarity of gene expression changes in TB in a cohort from Malawi and cohort from South Africa^25^. Increasing intensity of the red color indicates increase in disco.score and illustrates higher degree of similarity between gene expression in the two patient cohorts. Increasing intensity of the blue color indicates decrease in negative disco.score and a higher degree of dissimilarity in gene expression between two patient cohorts. (**D**) Log fold changes of gene expression of the cohort from South Africa plotted against log fold changes of gene expression of the cohort from Malawi for the genes belonging to module “Immune activation - generic cluster”, which was identified as concordant. The intensity of the color represents disco.score. Bars represent 95% confidence intervals (CI) for the log fold change.

We compiled lists of differentially expressed genes between WB transcriptome profiles of (i) TB patients and healthy controls; (ii) Mtb infected and uninfected 129S2 and C57BL/6 mice; and (iii) between transcriptional profiles of Mtb infected and uninfected human and murine macrophages (Table S1 and Fig. 1A). From this compilation we identified orthologous gene pairs between human and murine genes for each comparison. We included only the 1:1 orthologs (excluding potential in-paralogs) as defined by species interlinking in the Ensembl database, where homology predictions are generated by implementing maximum likelihood phylogenetic gene trees^24^.

To investigate the correlations in gene expression between human and murine data sets, we first applied the methods described in Seok *et al*.^4^ and Takao and Miyakawa^5^, as they use correlation, a common approach to assess similarity in gene expression regulation between species. We calculated the squared Pearson’s correlation coefficients (r^2^) of the maximum fold changes of all significantly differentially expressed gene pairs (p-value < 0.05)^4^. The obtained r² values indicated no significant correlation in the gene expression of human and murine whole blood or macrophage transcriptomic profiles upon Mtb infection (Table S1). Furthermore, we calculated Spearman’s rank correlation coefficients (r) for genes significantly regulated in both species in each comparison^5^. However, this criterion resulted in a minute number of genes included in the comparison, for example 316 out of 15,004 orthologous gene pairs in the comparison of C57BL/6 mouse WB data (on day 7 p.i.) versus human cohort from South Africa (Table S1, comparison 12)^25^. In summary, the previously described correlation-based approaches did not yield any valid conclusion regarding the relation of employed TB mouse models and human disease.

We therefore calculated GSE based on gene modules from Molecular Signatures Database (MSigDB)^26^, gene modules created by Chaussabel *et al*., Godec *et al*. and Li *et al*.^8,27,28^; the latter further referred to as “immune modules”) separately for mouse and human data sets. Even though we did not detect any significant correlation between the data sets, the enriched modules in both data sets were in large extent overlapping (Figure S2A; presented is the analysis with use of immune modules), indicating that there might be functionally related sets of genes regulated concordantly. However, this phenomenon could not be meaningfully analyzed statistically, due to heterogeneity of the data. Therefore, to quantify this effect, we developed a measure to assess the similarity of gene expression regulation for each orthologous gene pair in two data sets. The measure, discordance/concordance score (disco.score) is based on the following criteria of determining concordance: (i) magnitude of gene expression change (effect size), as well as (ii) significance and (iii) direction of gene expression change. The disco.score increases proportionally to both human and murine log_2_FC increase (or decreases analogously), increases with the decrease of summed p-values of genes in pair, and has negative sign if the expression change has opposite direction (Fig. 1B; for a general formula applicable to more than two heterologous data sets see Methods: “Disco.score calculation and GSE analysis”):

1 $$disco.score=\,{\mathrm{log}}_{2}\,F{C}_{Hs}\cdot {\mathrm{log}}_{2}\,F{C}_{Mm}\cdot |({\mathrm{log}}_{10}\,{P}_{Hs}+{\mathrm{log}}_{10}\,{P}_{Mm})|$$

where:

FC_Hs_ - fold change for human gene, as calculated in differential expression analysis

FC_Mm_ - fold change for murine gene, as calculated in differential expression analysis

P_Hs_ - p-value for human gene, as calculated in differential expression analysis

P_Mm_ - p-value for murine gene, as calculated in differential expression analysis

We then performed GSE on the list of genes from the two data sets ranked by decreasing or increasing disco.score (according to the algorithm presented on the Fig. 1A). We termed the modules enriched in the data set sorted by decreasing disco.score *concordant* and those enriched in data set sorted by increasing disco.score *discordant* (Figure S2B). Sorting the genes according to decreasing disco.score resulted in similar gene order as if they were sorted according to decreasing t-statistic, which depending on the method used to calculate differential expression is not always available, opposite to the universally used measure of gene expression change: log_2_FC and p-values (Figure S4).

We have thus created a measure of similarity of gene expression in heterologous data sets and an algorithm in which the similarity measurement is followed by GSE analysis to identify concordant and discordant elements of transcriptomic regulation.

### Validation tests

We used the 129S2 mouse WB data set as well as the human WB data set from The Gambia (including patients and healthy individuals) to validate the ability of disco.score for detecting concordant and discordant gene regulation between data sets from two different species. To this end, for different combinations of two parameters: number of genes in a module and percentage of significantly regulated genes in a module, we simulated a set of 100 concordant, 100 discordant and 100 random modules and compared the ability of disco.score to correctly detect the concordant and discordant ones. Indeed, the disco.score algorithm was able to correctly detect and classify the discordant and concordant modules (Figure S3; Methods: - Validation of disco.score with simulated modules).

To investigate how do the results of comparing data sets with disco.score reproduce a known biological background, we used disco.score to compare WB transcriptomic data sets from patients suffering of TB and sarcoidosis^29^. Previously published analysis showed that transcriptomic regulation from both disease groups was highly similar in comparison with healthy controls. Furthermore, analysis of genes in Kyoto Encyclopedia of Genes and Genomes (KEGG) pathways revealed similar differential expression patterns in TB and sarcoidosis, including genes involved in systemic lupus erythematosus, complement and coagulation cascades, toll-like receptor signaling, and Fc γ-receptor–mediated phagocytosis^29^. We calculated disco.score for each pair of corresponding genes from the two disease groups, resulting in 83% of genes having a positive disco.score. We then performed GSE on the list of genes ranked by decreasing and increasing disco.score, as well as separately for the TB and sarcoidosis patients. The identified gene sets were virtually identical in TB, sarcoidosis and modules identified after sorting the corresponding genes by decreasing disco.score (Figure S5A). To ascertain whether certain gene modules distinguished the two data sets quantitatively we analyzed GSE on the list of genes ranked according to increasing disco.score. As predicted we obtained no significant enrichment (Figure S5A). Next, we performed a similar validation test on the data set comparing similarities and differences in gene expression regulation among TB patients from Malawi and South Africa^25^. We plotted log_2_FC values of the Malawian against those of the South African cohort and calculated disco.score for each corresponding gene pair (Fig. 1C). As expected, the GSE in modules concordant among those two patient groups was abundant (94 concordant modules) and contained elements characteristic for the TB response. Only 4 modules were identified as discordant (Figure S5B; only the first 30 concordant modules and all the annotated discordant modules are shown). We also investigated the expression regulation of genes in each concordant module and observed that in accordance with assumptions of disco.score, the concordant modules consisted of genes regulated in the same directions and with similar magnitude (Fig. 1D).

Hence, the proposed algorithm for concordant and discordant gene modules identifies expected gene modules in data sets from cohorts characterized by similar transcriptome profiles.

### Validation of disco.score on human burn data set and the corresponding mouse model

We used disco.score to compare human and murine transcriptional responses to burn trauma, as previously compared in patients and C57BL/6J mice^4,5^. The result of Seok *et al*.^4^ indicated squared correlation coefficients between the data sets equal to 0.08. The group identified “Fc-γ Receptor-mediated Phagocytosis in Macrophage and Monocytes”, “IL-10 Signaling”, “Integrin Signaling”, “B cell receptor signaling” and “Toll-like receptor signaling” as the five most activated pathways in human burn and calculated r^2^ for the correlation of the five most regulated pathways between man and mouse which ranged from 0 to around 0.5. Takao & Miyakawa^5^ calculated the correlation coefficient between the same data sets equal to 0.68 because they excluded all the genes with log_2_FC <2 for man and log_2_FC <1.2 for mouse. They identified signaling pathways in which human and murine genes were regulated in the same direction, including “Innate immune response”, “Genes involved in Cytokine Signaling in Immune System” and “Lymphocyte Differentiation”.

We assigned the orthologs between human and murine genes from both data sets, separately in early response (time points up to 24 h post infection (p.i.)) and late response (time points between 24 h p.i. and 168 h p.i.), calculated the disco.score for each pair of orthologs and identified the concordant and discordant modules (Figure S6, for visualization purposes only 35 modules are shown) for early and late response according to the algorithm presented in Fig. 1A. At the first day p.i. there were 68 significant concordant modules between mouse and man, among which “immune activation – generic cluster” (LI.M37.0) was the most significant and “antigen presentation (lipids and proteins)” (LI.M28) had the largest effect size. Additionally, we detected two discordant modules: “NK cell surface signature” (LI.S1) and a non-annotated module (LI.M151, not shown). Many of the concordant modules were further related to B-cell signaling, T-cell signaling and innate immunity. However, one week after stimulation many of the T and B-cell related modules were still regulated in the human data set, but not in the murine one. Here, the concordant modules were related to innate immunity and cell metabolism. Intriguingly, the module “type I interferon response” (LI.M127) was at that time point highly significantly discordant between mouse and man (Figure S6).

In summary, our detection of concordant modules corresponded well with published findings^5^, which identified innate immune response related modules as similar in the human and murine response to burn. Our analysis indicated similar modules as highly regulated in humans but the coefficients of correlation with murine data were low. However, none of the previous studies noted the opposite gene expression change in NK (natural killer) cells or IFN (interferon) modules. Thus, disco.score followed by GSE identified concordances between human and murine burn data sets, which not only included previously described similarities but also indicated additional gene modules regulated in opposite manner between the two species which had not been identified previously.

### Disco.score identifies concordance and discordance of related human and murine data sets in TB

We acquired publicly available data sets from TB patients and murine TB and generated novel data sets from two models of TB, the low susceptible C57BL/6 strain and the highly susceptible strain 129S2. Analyzed data sets were derived from murine and human WB and macrophage samples (Table S1). We calculated differential expression of genes in each human and murine data set, calculated the disco.score for each pair of orthologous genes, identified concordant and discordant immune modules and MSigDB Hallmark Gene Sets (Figure S7)^26^ and verified concordance of gene expression change. The genes belonging to concordant modules were regulated in the same direction and the majority of them (for example 56% in comparison 2, Table S1) had non-negative weighed correlation coefficients. However, the modules comprising genes with negative correlation or correlation coefficient close to 0, but significantly regulated in the same direction in both mouse and man were also identified as concordant (in contrast to their assignment as ‘not concordant’ by correlation approach). In contrast, modules containing genes regulated in opposite direction but with positive correlation coefficient were discordant (Figure S1B and S2B). If not indicated otherwise, the results presented in the following text are based on transcriptomic modules created by Li *et al*.^27^. In conclusion, disco.score followed by GSE identified concordant and discordant gene expression changes between two mouse models and man more robustly than approaches based on correlation.

### Similarity of murine and human responses to infection changes over time

**Figure 2:**
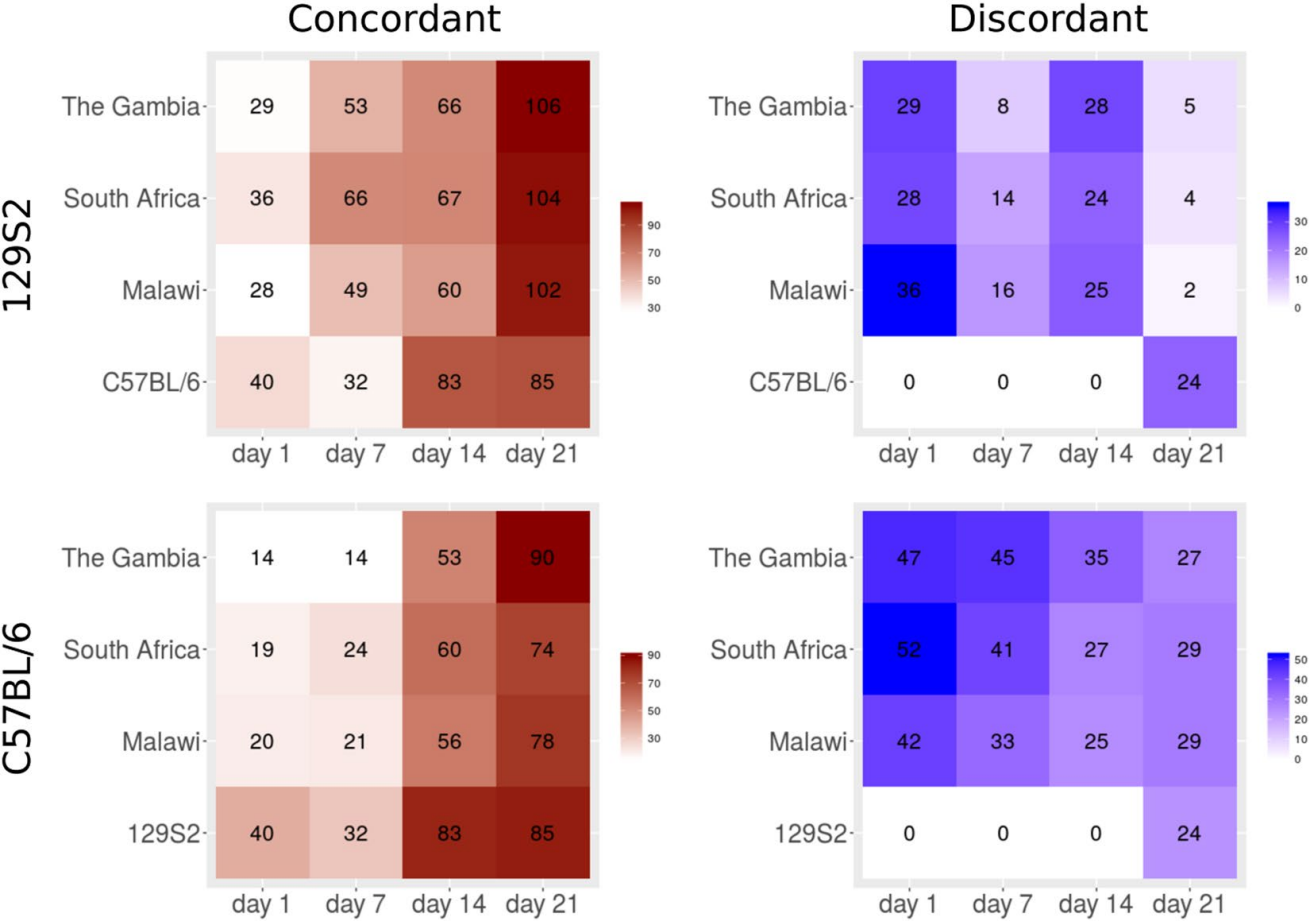
Module counts in comparisons of different human and mouse data sets. Red color refers to concordant and blue to discordant modules. **Upper panel**: Comparisons of 129S2 mouse strain data with human data sets from cohorts from The Gambia, South Africa, Malawi and with C57BL/6 mouse strain. **Lower panel**: Comparisons of C57BL/6 mouse strain data with human data sets from cohorts from Gambia, South Africa, Malawi and with 129S2 mouse strain.

We collected blood samples from 129S2 and C57BL/6 mouse strains before infection (day 0) as well as at time points 1, 7, 14 and 21 days p.i., performed microarray experiments and calculated differential gene expression comparing each time point p.i. with healthy controls (day 0). We then compared the time series data from both mouse strains with publicly available data sets from human cohorts from The Gambia^30^, South Africa and Malawi^25^. The human data sets included HIV negative (HIV−) TB patients in the three geographical locations, and HIV− and HIV+ individuals with latent TB infection (LTBI) from the same locations as controls. For each time point p.i. with Mtb in mice we identified concordant and discordant gene modules with respect to the human data sets from each of three populations and in comparison with the alternate mouse model. The highest number of concordant gene modules was found on day 21 p.i. in mice (Figure 2, upper panel). For both mouse strains, the degree of concordance with human data sets increased till day 21 p.i. (Figure 2). This can be partly explained by increasing numbers of differentially regulated genes with time p.i. In the two mouse strains, numbers of concordant modules increased from 40 at day 1 to above 80 at day 14 and day 21 p.i. The highest number of discordant modules between man and mice appeared on day 1 p.i. (29 modules), when the immune response to TB is not yet fully established. The number of discordances decreased towards day 21 p.i. in the comparison of human data with 129S2 mouse data. Notably, the discordant modules remained at a high level of around 40 modules in the comparison of human versus C57BL/6 strain. Consequently, there were no discordant gene modules in comparison between the two mouse strains up to day 14 p.i., whereas in the day 21 p.i. 24 modules containing genes regulated in opposite directions between the two strains were observed. In sum, although concordance between man and both mouse strains increased towards day 21 p.i., in C57BL/6 but not 129S2 mice discordances predominated.

### Discordance in 129S2 and C57BL/6 gene expression changes corresponds with the highly susceptible phenotype

After low dose aerosol Mtb infection, 129S2 mice suffer from progressive TB and succumb to disease within 40 days p.i., while C57BL/6 mice develop chronic TB and survive for more than 100 days p.i. Differences between the two strains are not visible at very early time points p.i., but between days 7–10 p.i. infiltration of inflammatory cells such as neutrophils occurs at the site of infection in 129S2 mice, while infiltration is not observed in the C57BL/6 mice until approx. day 14 p.i. At day 21 p.i. the 129S2 strain has already developed severe lung pathology characterized by large, necrotic lesions, whereas in C57BL/6 strain smaller, non-necrotic lesions developed with less inflammation.

We acquired data sets and compared the similarity of gene expression for both 129S2 and C57BL/6 mice. On day 1 p.i., the concordant modules between the two strains were related to innate immunity, immune signaling and platelets, with no discordant gene modules present (Figure S2C). On day 7 p.i., also spliceosome and proteasome related genes became co-regulated. On day 14, the amount of concordantly regulated modules increased further and we identified modules characteristic for TB, especially IFN related genes. On day 21 p.i. this set of modules remained concordant and complemented by cell cycle related modules.

Strikingly, at this time point several gene modules diverged in the two mouse strains with 13 T-cell related modules becoming discordant (Figure S2C, only modules with p-value for enrichment lower than 0.001 are shown). Note that the number of concordant modules between human and 129S2 mice exceeded the number of concordant modules between the two mouse models (Figure 2). The concordance between  the 129S2 mouse strain and human data sets encompassed “enriched in B cells” (LI.M47.0, LI.M47.1), “immune activation-generic cluster” (LI.M37.0), “antiviral IFN signature” (LI.M75), “enriched in neutrophils” (LI.M37.1) and “enriched in monocytes” (LI.M11.0). Discordant modules to C57BL/6 mice were similar in both human and 129S2 mice and encompassed “T cell activation and signaling” (LI.M5.1), “enriched in T cells” (LI.M7.0), “enriched in NK cells” (LI.M7.2). In summary, the T-cell response has been identified as the major difference between 129S2 and C57BL/6 mice at day 21 p.i., when the disease progressed more profoundly in the highly susceptible compared to the low susceptible mouse strain.

### T cell co-receptor genes drive the discordance between highly susceptible and low susceptible mice

**Figure 3:**
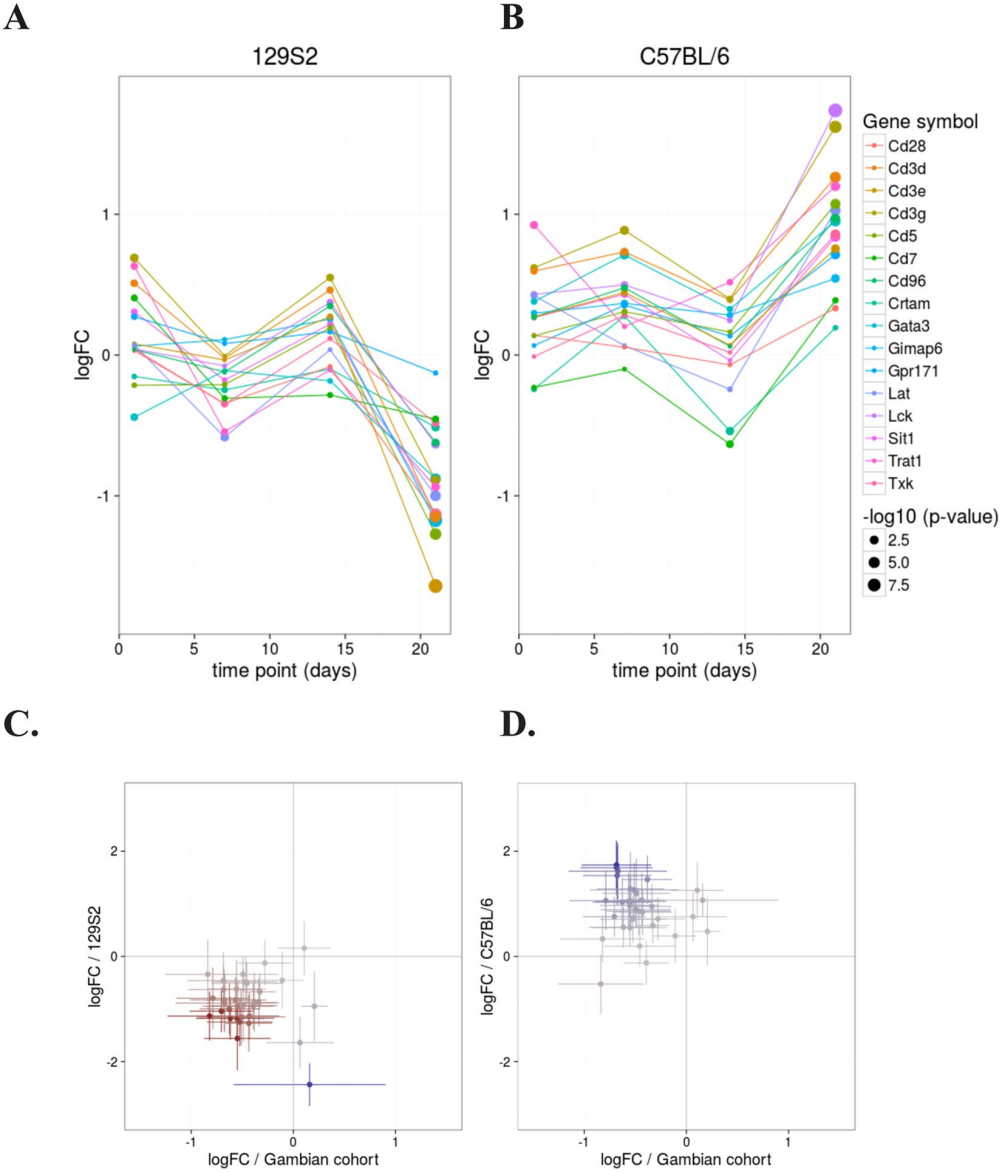
T-cell related genes with opposite expression pattern in 129S2 and C57BL/6 mouse strains. (**A**) Expression changes of selected genes belonging to the modules “enriched in T cells (**I**)”, “T cell activation and signaling” and “T cell activation (**I**)” at the time points day 1, day 7, day 14 and day 21 p.i. The selected genes drive the differences in the patterns of T-cell expression changes in 129S2 and C57BL/6 mice. (**B**) Log2FC of the set of 16 genes plotted for 129S2 or C57BL/6 mouse data vs. data from patient cohort from Gambia. Bars represent 95% confidence intervals (CI) for the log fold change.

We further investigated the expression of genes in the modules “enriched in T cells (I)” (LI.M7.0), “T cell activation and signaling” (LI.M5.1) and “T cell activation (I)” (LI.M7.1) in C57BL/6 and 129S2 mouse strains (Figure S8). Between day 14 and 21 p.i. genes were regulated in opposite manner in the two strains. The genes driving the opposite expression changes encoded mainly T-cell co-receptors (Cd28, Cd3d, Cd3e, Cd3g, Cd5, Cd7 and Cd96; Figure 3, upper panel, and Figure S9) which were up-regulated in the low susceptible strain C57BL/6 but down-regulated in the highly susceptible 129S2 strain. We investigated the expression changes of these genes in The Gambian cohort. The genes were highly concordantly regulated between the 129S2 mice and humans, and discordantly regulated between C57BL/6 mice and humans (Figure 3, lower panel). In this analysis we identified 16 genes related to T cell co-stimulation with opposite expression changes in human and highly susceptible 129S2 mice versus low susceptible C57BL/6 mice.

### Gene expression in response to Mtb infection is concordant in human and murine macrophages

We then analyzed transcriptome profiles of macrophages. We compared infected to non-infected human and murine (C57BL/6 derived) macrophages to test if the discordances found in blood are also present in gene expression profiles of macrophages. We calculated differential gene expression between human THP1 cells 6 h p.i. and uninfected cells in a data set collected in house and in a murine data set derived from a study by McNab *et al*.^31^ (Table S1, comparison 26). The disco.score analysis for each of the 13,881 orthologous gene pairs identified 34 concordant modules including “antiviral IFN signature” (LI.M75), “RIG-1 like receptor signaling” (LI.M68), “chemokine cluster (I)” (LI.M27.0) and others. Unlike in the case of WB comparisons, there were no enriched discordant modules in this comparison.

To determine whether the results are reproducible in other data sets derived from macrophages, we pursued the same strategy to distinguish concordant and discordant modules in other publicly available studies by Carow *et al*.^32^ (comparisons 25, 27, 29, 31 in Table S1) and Thuong *et al*.^33^ (comparisons 27–32 and 34–36 in Table S1). The human data set^33^ is derived from experiments performed with macrophages from patients who had recovered from pulmonary TB (samples referred to as PTB), TB meningitis (samples referred to as TBM) and individuals with LTBI. The macrophages were derived from isolated PBMCs and infected with Mtb for 4 h. We compared the LTBI samples (comparison 27), PTB samples (comparison 29) and TBM samples (comparison 31) with mouse bone marrow derived macrophages (BMDMs, Carow *et al*.^32^, non-stimulated and infected for 24 h with Mtb.

**Figure 4:**
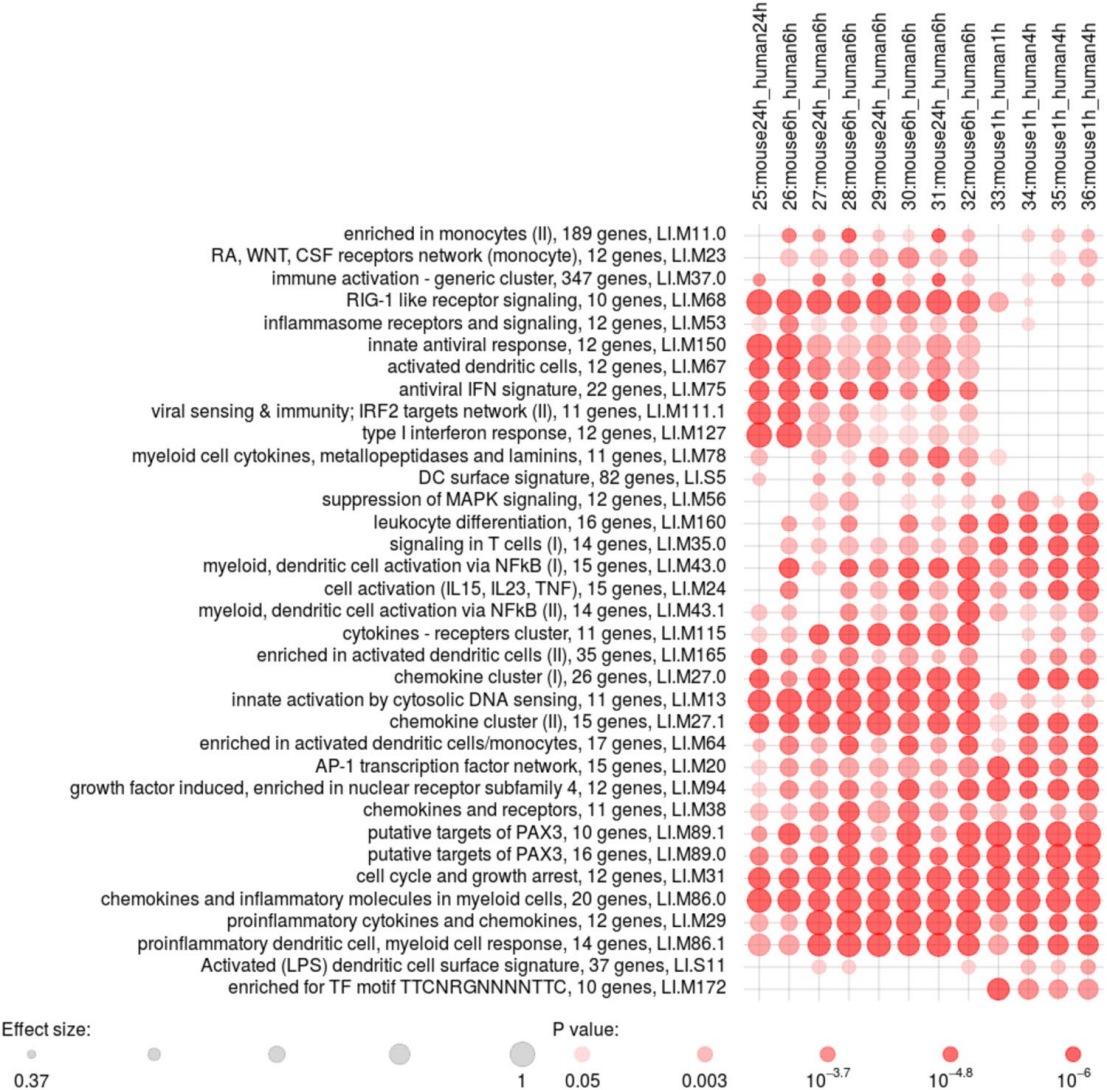
Gene expression patterns in human and mouse macrophages infected with Mtb. Concordant modules present among several comparisons of human and mouse macrophage data sets. P-value of module enrichment is illustrated by the intensity of the color and the effect size by the size of the dot. There were no overlapping concordant modules present in these comparisons. The modules are described by the titles followed by the original number of genes in module and ID. Module IDs correspond to modules IDs in R package *tmod* ^34^.

We identified 22 modules as concordant in each performed macrophage comparison (comparisons 25–36 in Table S1). They included the TB-characteristic IFN response related genes (LI.M75, LI.M127), chemokine clusters (LI.M27.0, LI.M27.1), “innate activation by cytosolic DNA sensing” (LI.M13), “cell cycle and growth arrest” (LI.M31) and “enriched in activated dendritic cells/monocytes” (LI.M64). No overlapping discordant modules emerged from the comparisons of different human and murine macrophage data sets (Figure 4). The early time points (1 h p.i., comparisons 33–36) contained 20 overlapping concordant and again no overlapping discordant modules. Most of the concordances were related to innate immunity (e.g. “proinflammatory cytokines and chemokines” (LI.M29), “cell activation (IL15, IL23, TNF)” (LI.M24), “myeloid, dendritic cell activation via NFkB (I)” (LI.M43.0), “chemokines and receptors” (LI.M38), whereas the IFN response was not apparent at this early time point.

The response to Mtb infection in blood cells focused on T- and B-cell signaling, whereas in macrophages the enrichment was mostly clustered in cell cycle, metabolic and innate immunity. Macrophages are critical elements of the first line of defense against infection^9^. Absence of discordant modules indicates that the expression regulation of the macrophage response to Mtb is largely conserved in mouse and man.

## Discussion

Our study identifies elements of the immune response to TB which are conserved or divergent between man and mouse. Intriguingly, we demonstrate that a highly susceptible mouse strain mimics more closely active TB in patients compared to a highly resistant strain. By implementing approaches to assess similarities between human and murine transcriptional data sets^4,5^ we determined correlations of murine and human gene expression changes upon Mtb infection and calculated enrichment in man-mouse gene modules^8^. None of the methods allowed specific distinction of similar (concordantly regulated) and dissimilar (discordantly regulated) elements of immunity. Rather, these techniques are restricted to identifying similarities or lack thereof.

Consequently, we created an algorithm to compare both similarities and dissimilarities of gene regulation in heterologous data sets based on disco.score, a measure of similarity of gene expression, complemented by GSE analysis. The method for identifying clusters of concordantly or discordantly regulated genes was validated on several data sets and applied to comparison of transcriptional responses in TB employing human and murine WB and monocytes (Table S1). We acquired publicly available data sets from human WB and macrophages and performed experiments to generate comparable data sets from murine macrophages and human macrophage-like THP-1 cells, as well as WB of 129S2 and C57BL/6 mouse strains which markedly differ in TB susceptibility.

Comparison of transcriptional profiles of low and highly susceptible mouse strains up to day 21 p.i. revealed increasing abundances of concordance and no discordance between the two strains. In striking contrast TB rapidly progressed in the highly susceptible strain after day 14 p.i. In parallel discordant modules emerged between low susceptible versus highly susceptible mice and TB patients.

At day 21 p.i. mouse transcriptional profiles presented the highest similarity with data sets from patient cohorts from different regions in Africa. Concordant modules included gene sets related to IFN response. Intriguingly, at day 21 p.i. very few modules between the highly susceptible mouse strain and TB patients were discordant, whereas a significant number of modules between the low susceptible strain and TB patients were discordant. Correspondingly, TB patients as well as highly susceptible mice 21 days p.i. suffered from active TB, while the low susceptible C57BL/6 mice showed no signs of symptomatic TB. We conclude that the transcriptional signature in 129S2 mice more closely reflects human TB pathology as compared to C57BL/6.

Discordances of either 129S2 murine or human data versus C57BL/6 mice were related to T cell functions. We identified a set of 16 genes involved in T cell related modules which were responsible for the discordance between 129S2 and human versus C57BL/6 gene expression. T cell proliferation is known to vary in lungs of susceptible mouse strain I/St and resistant mouse strain A/Sn, however the genes underlying those differences have not been identified^35^. Recently, differences in transcriptome regulation in peripheral blood leukocytes of susceptible and resistant lineages of *Macaca fascicularis* were described^36^. In TB-susceptible macaques down-regulation of T-cell related genes was observed at week 6 p.i., when animals had lost 10% body weight. Strikingly, the oppositely regulated genes in susceptible and resistant macaques included T-cell  co- receptors CD28 and CD3ε which were also discordant between susceptible and resistant mouse strains (Figure S9). It is therefore tempting to speculate that the 16 identified genes play a crucial role in acquired susceptibility and resistance in TB.

Genes related to innate immunity were largely concordant in human and murine transcriptomes, while discordant modules were overwhelmingly related to adaptive immunity, primarily T cells. At day 21 p.i. these discordances were present only in the low susceptible C57BL/6 mouse strain, which therefore continued to resemble individuals with persistent LTBI who do not progress to active disease.

Blood cell heterogeneity and variation in the composition of human and murine blood may have influenced the discrepancy found in gene expression regulation in WB of man and mouse. Another driving factor is the alteration in cell counts following infection. CD4+ and CD8+ cell counts are decreased in blood of TB patients compared to healthy individuals^13^. A study on gene expression in sorted CD4+ and CD8+ T cell populations from TB patients, LTBI and controls revealed a set of 33 differentially expressed diseasae associated candidate genes, which were enriched in JAK-STAT signaling pathway^37^. Enrichment in “Hallmark IL2 STAT5 signaling” gene set containing several of the identified genes was also concordant in comparisons of South African and murine WB data from C57BL/6 and 129S2 strains (Figure S7).

We demonstrate that disco.score identifies concordant and discordant gene modules between heterologous data sets. (i) This method can be applied to any two heterologous gene expression data sets, not necessarily of human or murine origin. (ii) It does not evaluate the overall similarity in the gene expression changes among all the genes in data sets. Rather, it distinguishes its concordant and discordant elements, based on the evolutionary principle that there are conserved elements of the immune system which can give a similar response to a particular challenge, and divergent ones which can react in unrelated manner and give rise to different phenotypes. (iii) The issue of arbitrary choice of genes included in the analysis is solved by performing the enrichment on the overall list of orthologous genes. (iv) It includes the possibility of using any gene module of interest, including well annotated immune modules^8,28,27^ which results in distinction of gene sets related to a specific immune function. (v) Disco.score is not restricted in particular value range, which allows direct comparison of results of different concordance analyses.

Disco.score provides measure of similarity of gene expression in two species. This measure has been used by us to sort the genes from most to least concordant for the purpose of performing modular analysis to identify similar and different elements of immune response between mouse and man. However, this is not the only type of analysis one can perform using disco.score. For example, if the focus of a study is a particular gene or a group of genes, disco.score enables comparison of the expression pattern of this gene between different organisms over different conditions. It also allows the creation of novel modules –e.g. top ranking genes in disco.score can be combined in a new module characterizing a particular disease model. The outcome of our method highly depends on the quality of microarray data as well as their annotations. At a given time the algorithm compares two data sets only and does not allow correction for cell numbers which influence interpretation of the transcriptional studies and should be performed independently.

We conclude that gene expression change at day 21 p.i. in WB of highly susceptible 129S2 mice, but not low susceptible C57BL/6 mice, closely mimics that in blood of TB patients. Therefore, depending on the mouse model used, gene expression may variably mirror human disease. This needs to be taken into account when planning experiments with translatability to human TB, e.g. vaccination studies. In contrast to WB, gene expression in murine macrophages corresponded well with that in human macrophages.

Our study comparing two different murine models of TB emphasizes a general need to identify the best-fit animal model as correlate for a particular human disease. Our straightforward approach is a first step towards this direction and shows promise in revealing which is which: species-specific responses which may not be translatable from the model organism to human, or widely conserved processes for which the model organism provides a robust approximation to human.

## Methods

### Mice and Mtb infection

129S2 (129SvPas) mice were bred and kept under specific pathogen-free (SPF) conditions at the Max Planck Institute for Infection Biology in Berlin, Germany. C57BL/6 animals were purchased from Charles Rivers Laboratories. Mice were matched for age and sex, and co-housed for at least two weeks under specific pathogen-free (SPF) conditions at the Max Planck Institute for Infection Biology in Berlin, Germany before start of the experiments. At the time of infection, all mice were 9–12 weeks of age. Aerosol infection with Mtb strain H37Rv and enumeration of bacteria in lung tissue were performed as previously described^15^. All experiments were approved by the State Office for Health and Social Affairs (Landesamt fuer Gesundheit und Soziales) and conducted in accordance with German Animal Protection Law.

### Blood collection and RNA isolation

At indicated time points mice were anesthetized by intraperitoneal injection of 16 mg/kg bodyweight Rompun and 120 mg/kg bodyweight Ketavet in PBS. Blood was drawn from the inferior vena cava of all mice using a 26 G needle. 200 μl of blood were directly transferred into 800 μl of TRIzol® (Invitrogen). Total RNA extraction of all blood samples was performed according to the manufacturer’s instructions. The RNA yield and A260/280 ratio were measured with a NanoDrop ND 100 spectrometer (NanoDrop Technologies), and RNA integrity was verified using an 2100 Bioanalyzer (Agilent Technologies) with a RNA integrity number (RIN) higher than 7.

### Microarrays

Total RNA of blood samples was labeled with the Low Input Quick Amp Labeling (Agilent Technologies) according to manufacturer’s instructions. Quantity and labeling efficiency were verified before hybridization of the samples to SurePrint G3 Mouse GE 8 × 60 K Microarray (Agilent Technologies, Product Number G4852A, Design ID 028005). Scanning of microarrays was performed with 3 μm resolution using a high-resolution laser microarray scanner (Agilent Technologies G2565CA). Quality, reproducibility and reliability of single microarray data was accessed by the 1-color gene expression QC report from Agilent Technologies.

### Acquisition of THP1 data

The human monocytic cell line THP-1 (ATCC TIB-202) was maintained in RPMI 1640 (Gibco), supplemented with 10% (v/v) heat-inactivated fetal calf serum (Gibco), 1% (v/v) penicillin–streptomycin (Gibco), 1% (v/v) L-glutamine (Gibco), 1% (v/v) HEPES buffer (Gibco) and 0.05 M 2-mercaptoethanol (Gibco). Cells were differentiated into macrophages by treatment with 50 ng/ml of phorbol 12-myristate 13-acetate (PMA, Calbiochem). Subsequently they were rested for 48 hours and afterwards infected with single-bacterial suspensions of the virulent strain H37Rv, at a multiplicity of infection of 5. At 1, 6 and 24 h following infection, macrophages were lysed with 4 M guanidine isothiocyanate solution (Invitrogen), eukaryotic RNA was stabilized in Trizol LS (Invitrogen) and extracted according to vendor’s instructions. The RNA yield was detected with a NanoDrop ND 100 spectrometer (NanoDrop Technologies), and RNA integrity was estimated using the 2100 Bioanalyzer (Agilent Technologies).

### Macrophage RNA microarrays

Total RNA of infected and uninfected THP-1 control cells was labeled with the Quick Amp Labeling (Agilent Technologies) according to manufacturer’s instructions. After quality and labeling efficiency control samples were hybridized to 4 × 44 K Whole Human Genome Microarray kits (Agilent Technologies, Product Number G4112F, Design ID 014850). Scanning of microarrays was performed with 5 μm resolution using a G2565CA high-resolution laser microarray scanner (Agilent Technologies) using extended dynamic range (XDR). Raw microarray data were extracted with the Agilent FE software V10.5.1.1. and GE1_105_Dec08 protocol using default settings.

### Data preprocessing

Data analysis was performed in R version 3.2.3 (2015-12-10), and a script including all analytical steps is available upon request. External microarray data sets have been downloaded from Gene Expression Omnibus database using R package GEOQuery (ref.^39^; GSE34608, GSE19491, GSE3284, GSE47673, GSE23508, GSE11199, GSE37250). Data sets obtained in this study have been uploaded to GEO under accession ID GSE89392 (link for the reviewers: https://www.ncbi.nlm.nih.gov/geo/query/acc.cgi?token=yrkpgamgzbcdlaz&acc=GSE89392). The data sets have been analyzed with limma R package for differential expression analysis^40^. The data sets were background corrected using the normexp method and quantile normalized between arrays. Limma *lmFit* function was used to fit linear models which included the factors: stimulus type and time point. The p-values were calculated based on the moderated t-statistic.

### Ortholog assignment

Orthologous genes were assigned to each other between corresponding human and mouse data sets used in each comparison. Probe names specific to the microarray used were assigned an ENSEMBL identifier with use of “mapIds” function from biomaRt package (version 2.24.1)^41,42^. Multiple repeating probes were averaged by applying *limma* “avereps” function. Then, orthologous human and mouse genes were identified with biomaRt “getLDS” function based on homology mapping between different species interlinked in Ensembl data base (with attributes and filters defined as “ensembl_gene_id”). Only the putative orthologs with a 1:1 mapping (no potential in-paralogs) were included in the further analysis.

### Disco.score calculation and GSE analysis

We have created an R-package *disco* for identification and visualization of concordant and discordant gene modules. The *disco* package is available on CRAN (http://cran.r-project.org/web/packages/disco/) Disco score was calculated for each pair of orthologous genes using *discoScore* function. Concordantly and discordantly regulated gene sets were identified by performing gene set enrichment analysis with R-package *tmod* (version 0.27^43^) using CERNO statistical test, which is a variant of Fisher’s method adapted to gene set enrichment analysis^44^ on the list of genes sorted by the decreasing or increasing disco.score, respectively. Disco.score for particular genes has been visualized with the color gradient on the plots presenting log_2_ of fold change of gene expression in stimulated versus non-stimulated organisms.

The general formula for disco score applicable to n data sets is defined by the equation:

2 $$disco.score:=-\sum _{i=1}^{n-1}\sum _{j=i+1}^{n}{\mathrm{log}}_{2}\,F{C}_{i}\cdot {\mathrm{log}}_{2}\,F{C}_{j}\cdot ({\mathrm{log}}_{10}\,{P}_{i}\,+{\mathrm{log}}_{10}\,{P}_{j})$$

where:

n - number of data sets analyzed

FC_i_ - fold change for gene in the data set i, as calculated in differential expression analysis

FC_j_ - fold change for gene in the data set j, as calculated in differential expression analysis

P_i_ - p-value for human gene in the data set i, as calculated in differential expression analysis

P_j_ - p-value for murine gene in the data set j, as calculated in differential expression analysis

The method and examplary result of the comparison of three data sets with disco.score is illustrated in the Figure S9.

### Validation of disco.score with simulated modules

We have used the human data set from the Gambia and mouse data set 21 days p.i. (129S2 mice) and a simulated set of modules to test the performance of disco.score algorithm in retrieving concordantly and discordantly regulated modules. We used the existing murine and human data sets, but we have simulated the assignment of genes to gene sets, thus defining a priori which gene sets contain concordant genes, which gene sets contain discordant genes, and which are negative controls. We then tested whether the disco algorithm is able to detect these a priori defined gene sets.

We have simulated gene sets containing 10, 20, 30, 40 or 50 genes, out of which 10%, 20% or 30% were either concordantly regulated or discordantly regulated (Table S2). In addition, we have generated modules consisting of 10 to 50 genes, which contained equal number of either concordantly or discordantly regulated genes. Each parameter combination (number of genes, number of regulated genes, type of regulation: concordant, discordant or equal number) has been replicated 100 times; an equal number of 100 replicates of a suitable negative control modules was then added to the superset. For concordant modules, the control modules contained only non-concordant genes (including discordant genes, non-regulated genes or genes with significant differences only in one organism); for discordant, only non-discordant genes; for equal number, only genes that were neither concordant nor discordant.

Next, with each set of 200 modules (out of which 100 were concordant or discordant and 100 were negative controls) we performed CERNO test on the list of genes sorted by disco.score and identified the concordant and discordant modules. Then, we sorted the detected modules according to the p-values for enrichment and calculated area under curve (AUC) that corresponds to how accurately the algorithm detected the concordant or discordant modules (Figure S3).

### Positive controls

We used a data set published by Maertzdorf *et al*.^30^ containing whole blood expression profiles from patients suffering from TB and sarcoidosis and healthy controls as positive controls for disco.score. The two diseases give expression profiles undistinguishable from each other when compared to healthy controls. We calculated differential expression between the 18 sarcoidosis patients and 18 healthy controls and between the 8 TB patients and 18 healthy controls. We matched the genes between both groups and calculated disco.score for each pair of corresponding human genes. We then sorted the list of differentially expressed genes by decreasing disco.score and performed gene set enrichment analysis to distinguish concordant gene modules between the two groups. Then we sorted the gene list according to increasing value of the disco.score and performed gene set enrichment analysis to distinguish discordant gene modules.

### Data availability statement

The datasets generated during and/or analyzed during the current study are available in the Gene Expression Omnibus repository under accession ID GSE89392 (link for the reviewers: https://www.ncbi.nlm.nih.gov/geo/query/acc.cgi?token=yrkpgamgzbcdlaz&acc=GSE89392). The created R-package *disco* is available on CRAN, under the link: http://cran.r-project.org/web/packages/disco/.

## Electronic supplementary material

Supplementary material
