# Supplementary material for: Concordant and discordant gene expression patterns in mouse strains identify best-fit animal model for human tuberculosis
Source: Sci Rep. 2017 Sep 21;7:12094. doi: 10.1038/s41598-017-11812-x (PMC5608750; doi:10.1038/s41598-017-11812-x)
Supplement: Supplementary file 1 — Supplementary material [file 41598_2017_11812_MOESM1_ESM.pdf]

Supplementary Material

Concordant and discordant gene expression patterns in mouse strains identify best-fit animal model for human tuberculosis

Teresa Domaszewska, Lisa Scheuermann, Karin Hahnke, Hans Mollenkopf, Anca Dorhoi, Stefan H.E. Kaufmann, January Weiner

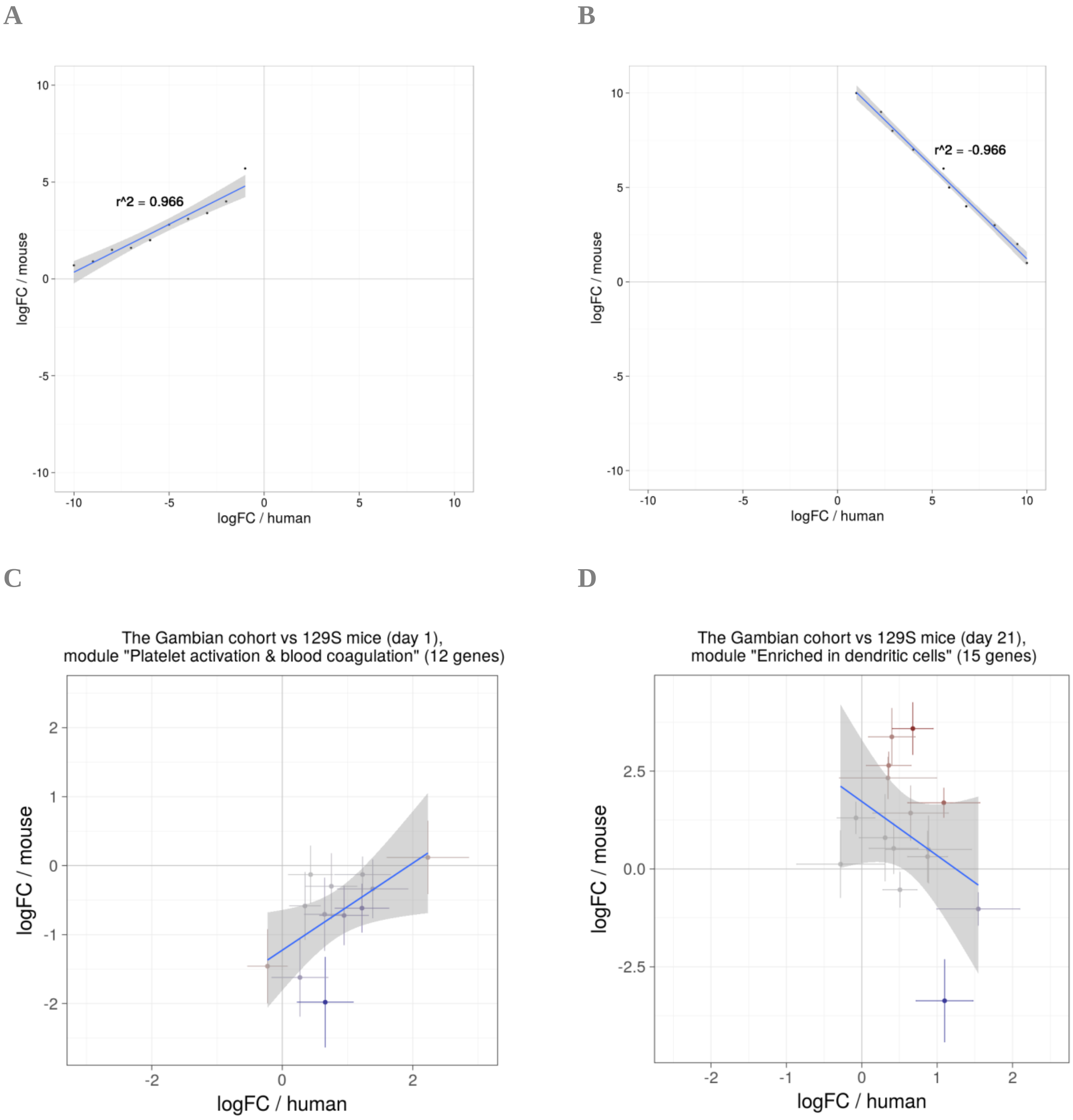

Figure S1 Correlation can mistakenly identify gene sets as concordant or discordant

The blue line represents linear model fit into the data; grey shade represents confidence intervals for the linear model. In the lower panel, the intensity of the color represents disco.score value. Bars represent 95% confidence intervals (CI) for the log fold change.

(A) Theoretical example demonstrating a positive correlation coefficient r^2 even though the group of genes in two data sets is regulated in opposite directions in both species. (**B)** Theoretical example demonstrating that the correlation coefficient r^2 can be negative even though the group of genes in the two data sets is regulated in the same directions in both species. (C) Log fold changes of gene expression in 129S2 strain 1 day after infection plotted against log fold changes of the cohort from Gambia for the genes belonging to module “Platelet activation and blood coagulation”, which was identified as discordant even though its correlation coefficient r is significantly positive and equals 0.617 (p-value 0.033). (**D)** Log fold changes of gene expression in 129S strain 21 days after Mtb infection plotted against log fold changes of the cohort from Gambia for the genes belonging to module “Type I interferon response”, which was identified as concordant even though the correlation coefficient r was significantly negative and equaled -0.816 (p-value 0.025).

A

Modules enriched in different data sets

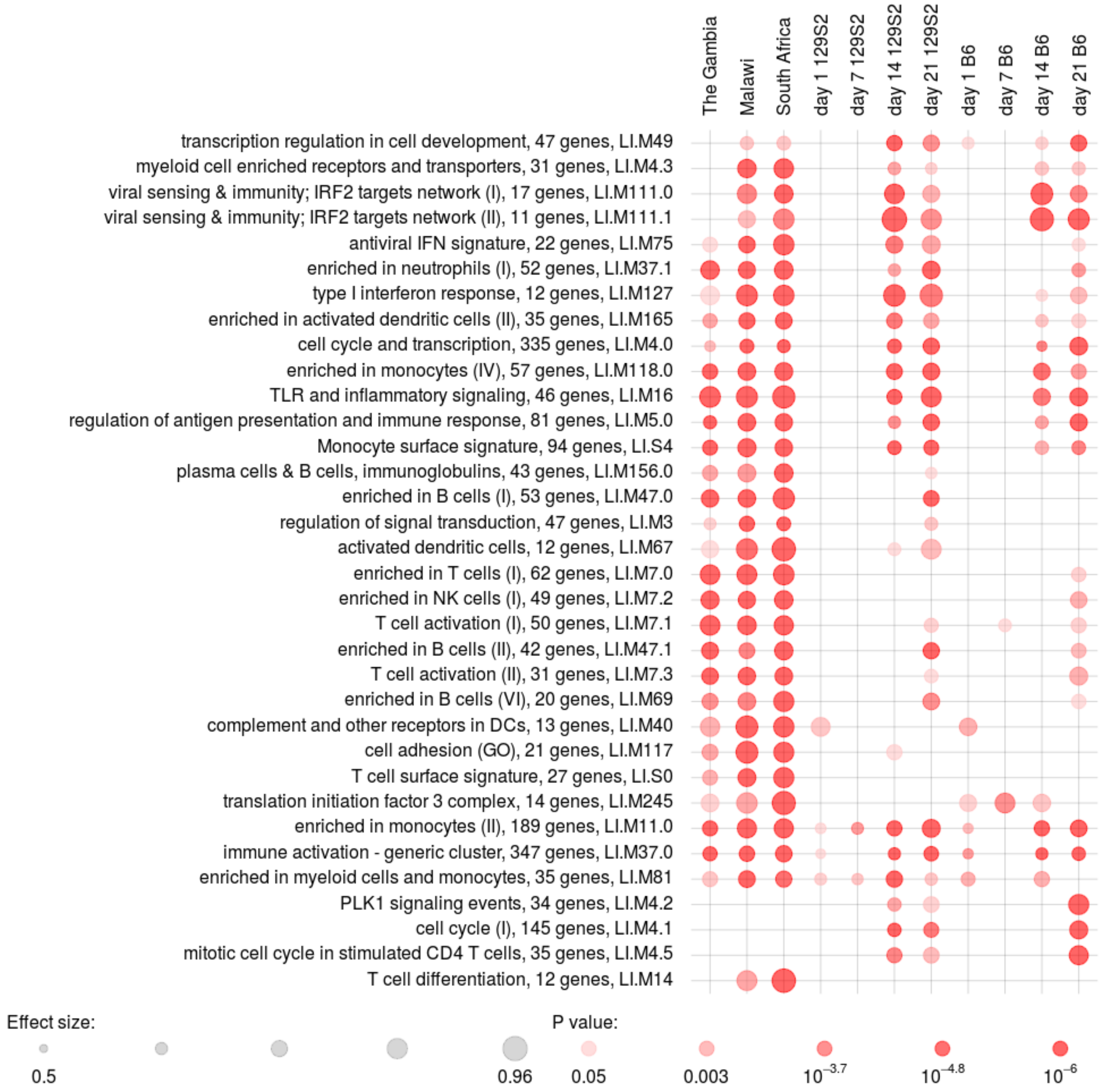

The Gambia
Malawi
South Africa
day 1 129S2
day 7 129S2
day 14 129S2
day 21 129S2
day 1 B6
day 7 B6
day 14 B6
day 21 B6
transcription regulation in cell development, 47 genes, LI.M49
myeloid cell enriched receptors and transporters, 31 genes, LI.M4.3
viral sensing & immunity; IRF2 targets network (I), 17 genes, LI.M111.0
viral sensing & immunity; IRF2 targets network (II), 11 genes, LI.M111.1
antiviral IFN signature, 22 genes, LI.M75
enriched in neutrophils (I), 52 genes, LI.M37.1
type I interferon response, 12 genes, LI.M127
enriched in activated dendritic cells (II), 35 genes, LI.M165
cell cycle and transcription, 335 genes, LI.M4.0
enriched in monocytes (IV), 57 genes, LI.M118.0
TLR and inflammatory signaling, 46 genes, LI.M16
regulation of antigen presentation and immune response, 81 genes, LI.M5.0
Monocyte surface signature, 94 genes, LI.S4
plasma cells & B cells, immunoglobulins, 43 genes, LI.M156.0
enriched in B cells (I), 53 genes, LI.M47.0
regulation of signal transduction, 47 genes, LI.M3
activated dendritic cells, 12 genes, LI.M67
enriched in T cells (I), 62 genes, LI.M7.0
enriched in NK cells (I), 49 genes, LI.M7.2
T cell activation (I), 50 genes, LI.M7.1
enriched in B cells (II), 42 genes, LI.M47.1
T cell activation (II), 31 genes, LI.M7.3
enriched in B cells (VI), 20 genes, LI.M69
complement and other receptors in DCs, 13 genes, LI.M40
cell adhesion (GO), 21 genes, LI.M117
T cell surface signature, 27 genes, LI.S0
translation initiation factor 3 complex, 14 genes, LI.M245
enriched in monocytes (II), 189 genes, LI.M11.0
immune activation - generic cluster, 347 genes, LI.M37.0
enriched in myeloid cells and monocytes, 35 genes, LI.M81
PLK1 signaling events, 34 genes, LI.M4.2
cell cycle (I), 145 genes, LI.M4.1
mitotic cell cycle in stimulated CD4 T cells, 35 genes, LI.M4.5
T cell differentiation, 12 genes, LI.M14
Effect size:
0.5
0.96
P value:
0.05
0.003
10^-3.7
10^-4.8
10^-6

B

Concordant modules in comparisons of 129S2 WB from different time points

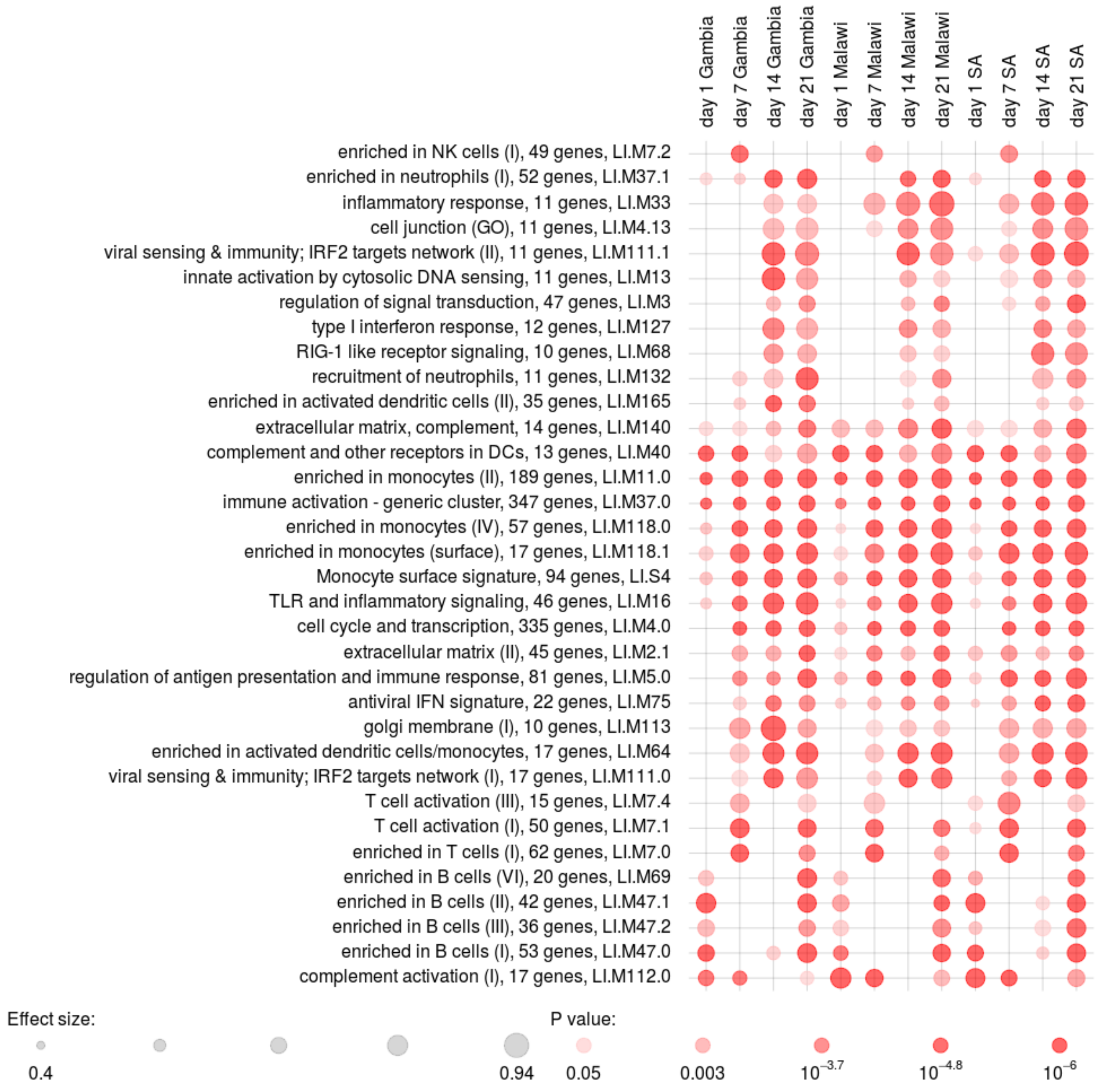

Concordant modules in comparisons of C57BL/6 WB from different time points

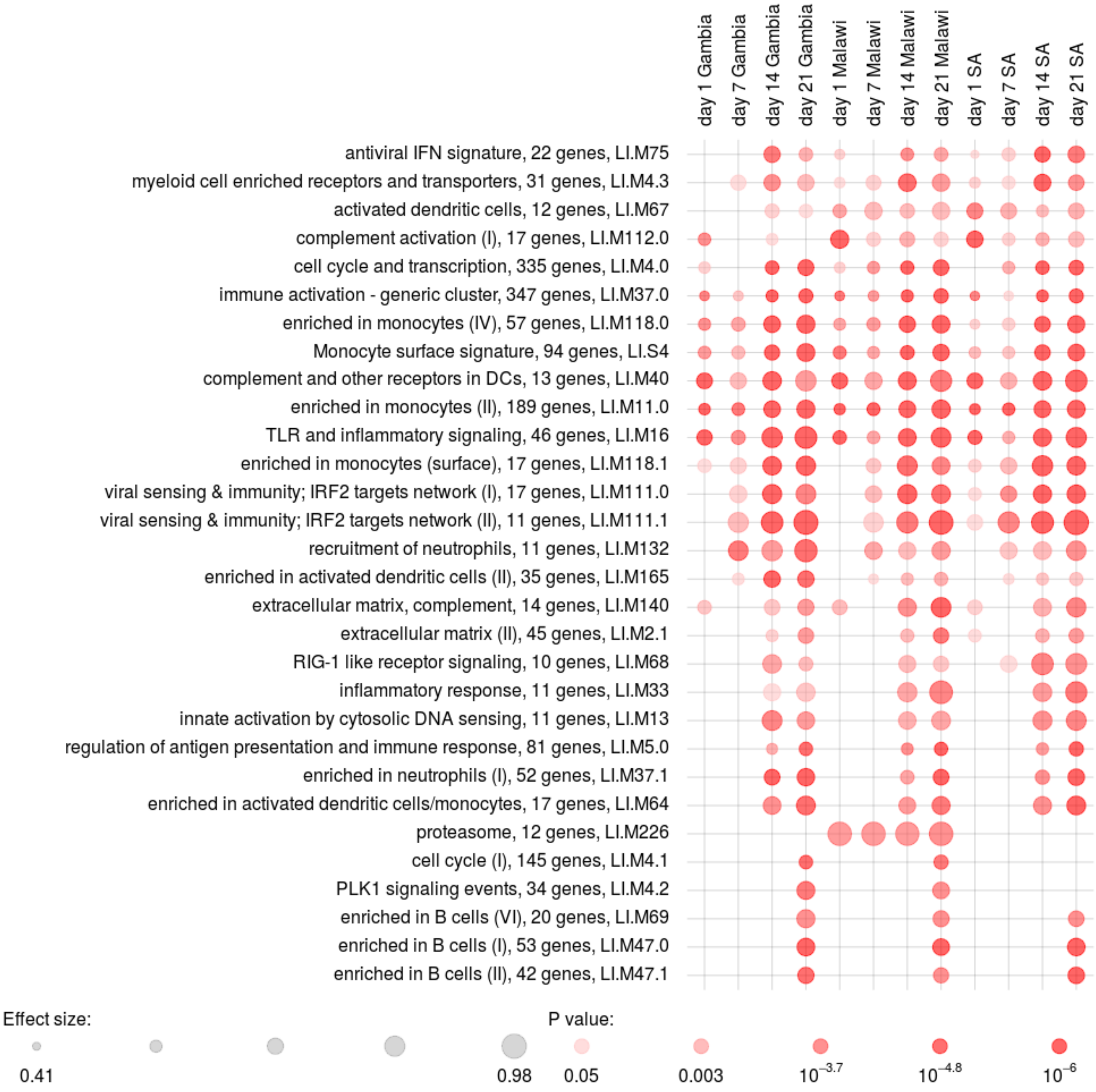

Discordant modules in comparisons of 129S2 WB from different time points

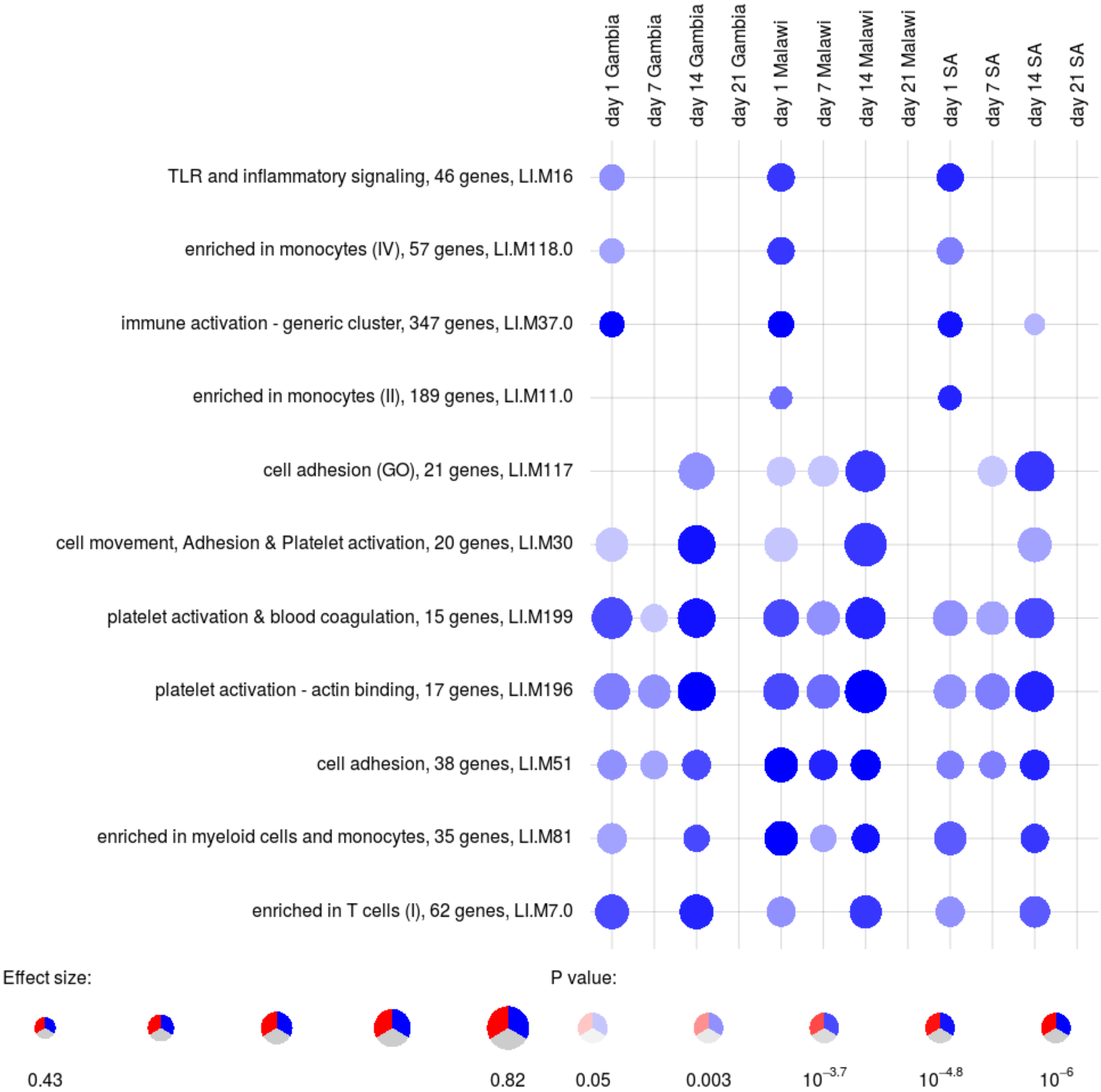

Discordant modules in comparisons of C57BL/6 WB from different time points

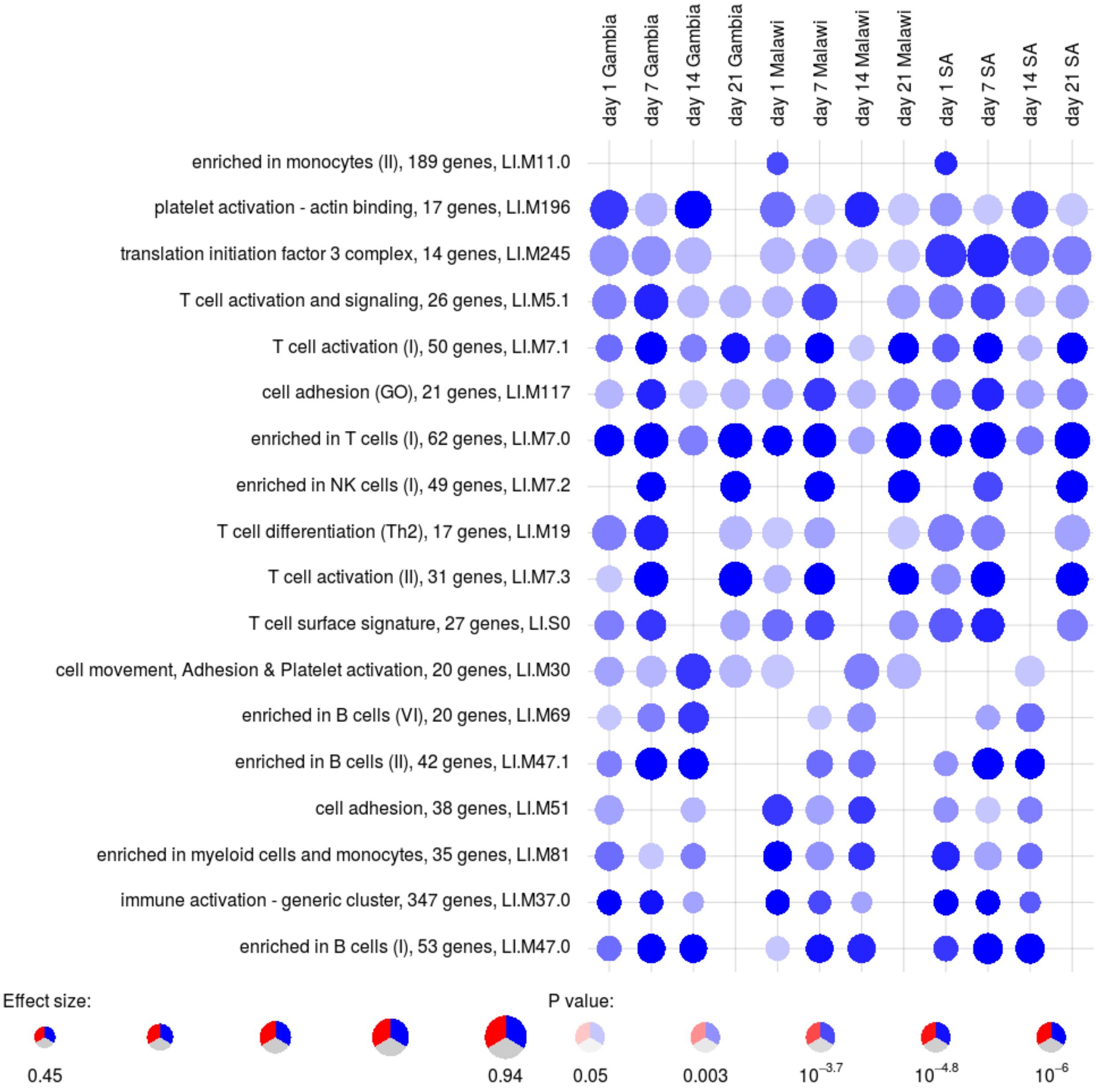

C

Concordant and discordant modules in comparison of two mice strains along time

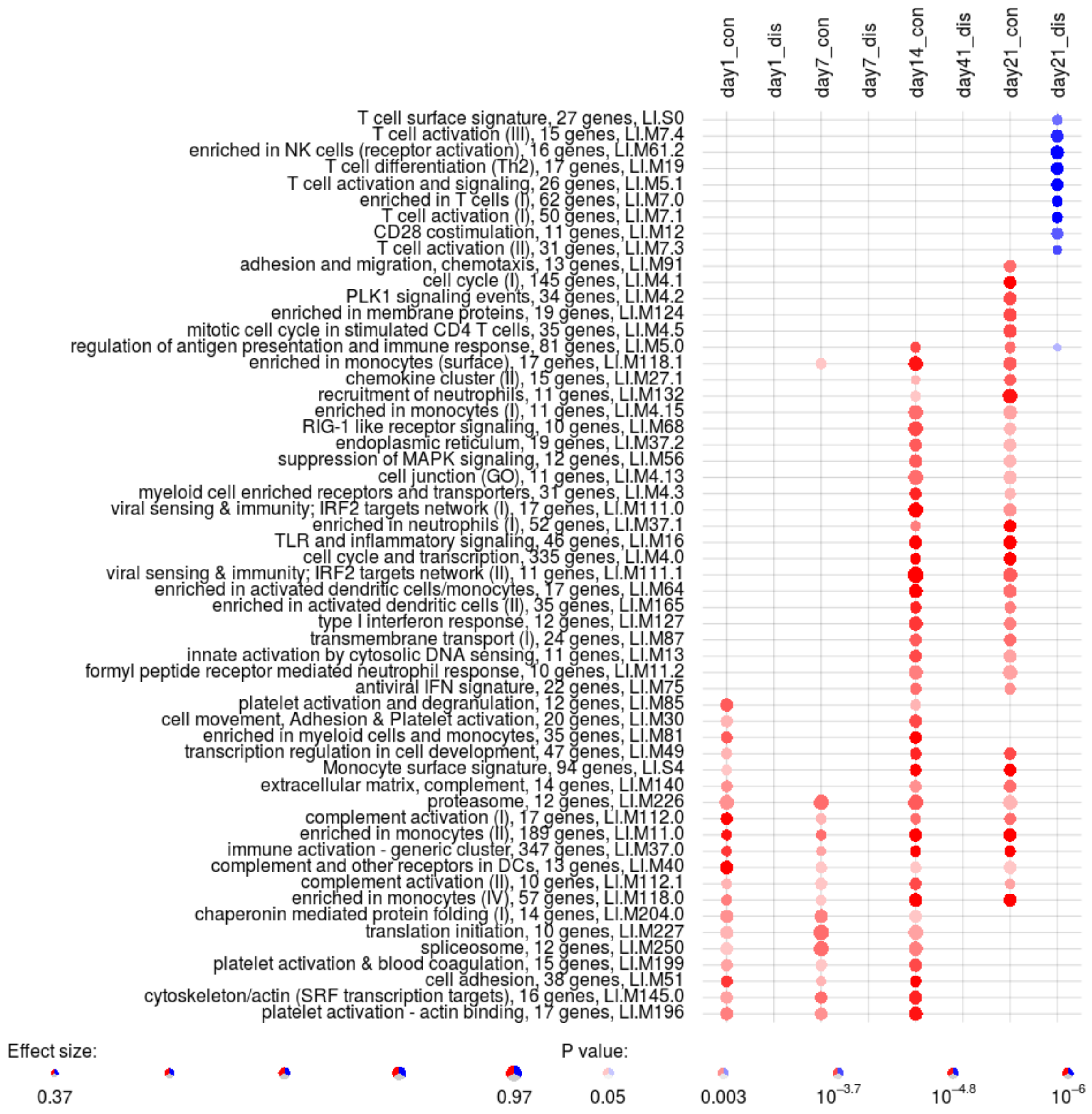

Figure S2 Gene set enrichment in human and murine data sets

(A) Modules enriched in human WB data sets from Gambia, Malawi and South Africa (labeled "SA") and WB data sets from C57BL/6 and 129S2 mice in time points: day 1, day 7, day 14, day 21 p.i.. **(B)** Concordant (red) and discordant (blue) gene modules identified with disco.score among the species. P-value is illustrated by the intensity of the color and the effect size by the size of the dot, as presented in the legend under the first plot. Only the modules with p-value for the enrichment lower than 0.005 are shown. **(C)** Concordant (red) and discordant (blue) gene modules identified with disco.score among the 129S2 and C57/BL6 mouse strains. P-value is illustrated by the intensity of the color and the effect size by the size of the dot, as presented in the legend under the first plot. Only the modules with p-value for the enrichment lower than 0.001 are shown. The modules are described by the titles followed by the original number of genes in module and ID**.**

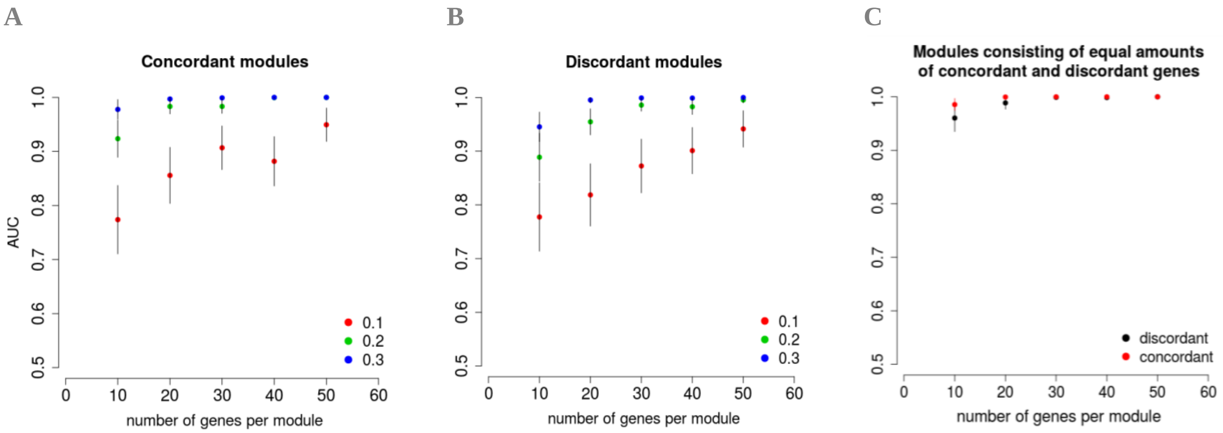

Figure S3 Simulation results

Accuracy of the detection of **(A)** concordant modules, **(B)** discordant modules, and **(C)** modules concordant and discordant at the same, time illustrated by AUC corresponding to different numbers of genes in the modules and different percentage of regulated genes in the modules.

A

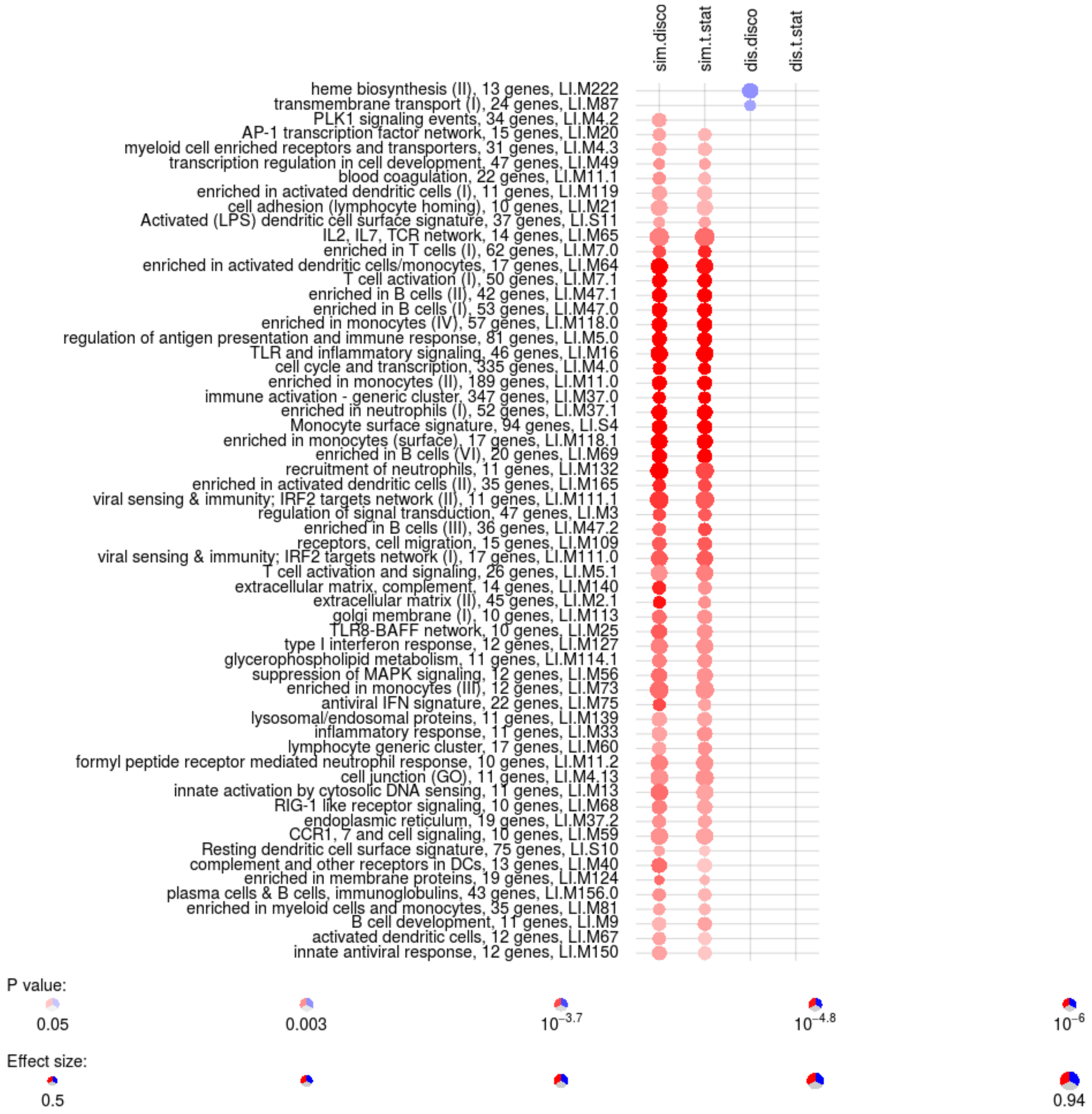
sim.disco
sim.t.stat
dis.disco
dis.t.stat
heme biosynthesis (II), 13 genes, LI.M222
transmembrane transport (I), 24 genes, LI.M87
PLK1 signaling events, 34 genes, LI.M4.2
AP-1 transcription factor network, 15 genes, LI.M20
myeloid cell enriched receptors and transporters, 31 genes, LI.M4.3
transcription regulation in cell development, 47 genes, LI.M49
blood coagulation, 22 genes, LI.M11.1
enriched in activated dendritic cells (I), 11 genes, LI.M119
cell adhesion (lymphocyte homing), 10 genes, LI.M21
Activated (LPS) dendritic cell surface signature, 37 genes, LI.S11
IL2, IL7, TCR network, 14 genes, LI.M65
enriched in T cells (I), 62 genes, LI.M7.0
enriched in activated dendritic cells/monocytes, 17 genes, LI.M64
T cell activation (I), 50 genes, LI.M7.1
enriched in B cells (II), 42 genes, LI.M47.1
enriched in B cells (I), 53 genes, LI.M47.0
enriched in monocytes (IV), 57 genes, LI.M118.0
regulation of antigen presentation and immune response, 81 genes, LI.M5.0
TLR and inflammatory signaling, 46 genes, LI.M16
cell cycle and transcription, 335 genes, LI.M4.0
enriched in monocytes (II), 189 genes, LI.M11.0
immune activation - generic cluster, 347 genes, LI.M37.0
enriched in neutrophils (I), 52 genes, LI.M37.1
Monocyte surface signature, 94 genes, LI.S4
enriched in monocytes (surface), 17 genes, LI.M118.1
enriched in B cells (VI), 20 genes, LI.M69
recruitment of neutrophils, 11 genes, LI.M132
enriched in activated dendritic cells (II), 35 genes, LI.M165
viral sensing & immunity; IRF2 targets network (II), 11 genes, LI.M111.1
regulation of signal transduction, 47 genes, LI.M3
enriched in B cells (III), 36 genes, LI.M47.2
receptors, cell migration, 15 genes, LI.M109
viral sensing & immunity; IRF2 targets network (I), 17 genes, LI.M111.0
T cell activation and signaling, 26 genes, LI.M5.1
extracellular matrix, complement, 14 genes, LI.M140
extracellular matrix (II), 45 genes, LI.M2.1
golgi membrane (I), 10 genes, LI.M113
TLR8-BAFF network, 10 genes, LI.M25
type I interferon response, 12 genes, LI.M127
glycerophospholipid metabolism, 11 genes, LI.M114.1
suppression of MAPK signaling, 12 genes, LI.M56
enriched in monocytes (III), 12 genes, LI.M73
antiviral IFN signature, 22 genes, LI.M75
lysosomal/endosomal proteins, 11 genes, LI.M139
inflammatory response, 11 genes, LI.M33
lymphocyte generic cluster, 17 genes, LI.M60
formyl peptide receptor mediated neutrophil response, 10 genes, LI.M11.2
cell junction (GO), 11 genes, LI.M4.13
innate activation by cytosolic DNA sensing, 11 genes, LI.M13
RIG-1 like receptor signaling, 10 genes, LI.M68
endoplasmic reticulum, 19 genes, LI.M37.2
CCR1, 7 and cell signaling, 10 genes, LI.M59
Resting dendritic cell surface signature, 75 genes, LI.S10
complement and other receptors in DCs, 13 genes, LI.M40
enriched in membrane proteins, 19 genes, LI.M124
plasma cells & B cells, immunoglobulins, 43 genes, LI.M156.0
enriched in myeloid cells and monocytes, 35 genes, LI.M81
B cell development, 11 genes, LI.M9
activated dendritic cells, 12 genes, LI.M67
innate antiviral response, 12 genes, LI.M150
P value:
0.05
0.003
10−3.7
10−4.8
10−6
Effect size:
0.5
0.94

B

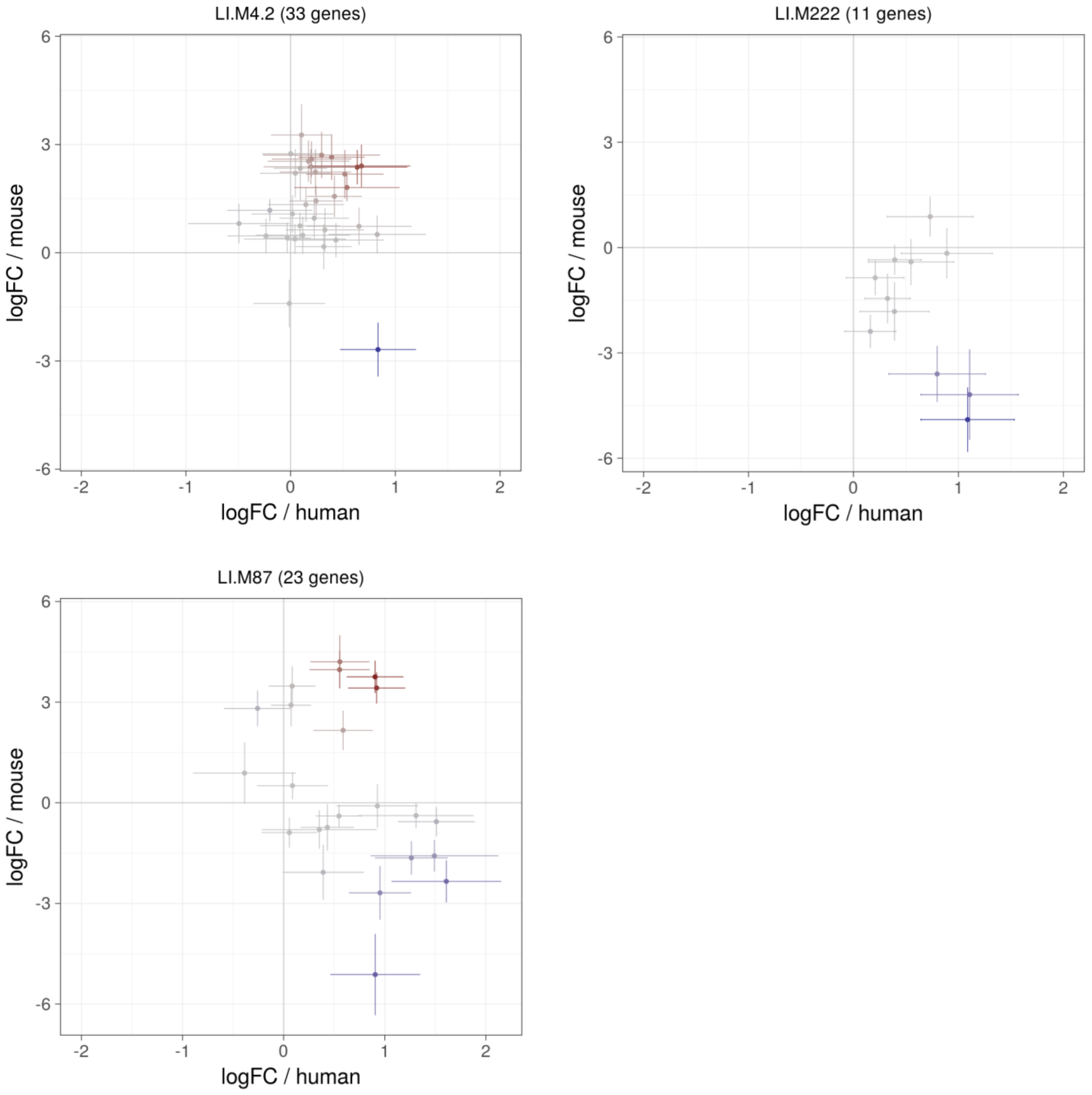

Figure S4 Sorting genes by disco.score results in more sensitive concordance and discordance detection compared with t-statistic

(A) Concordant (red) and discordant (blue) modules enriched in comparison of WB expression profiles of patients from Gambia and 129S2 mice at day 21 p.i. detected with disco.score and on the basis of t-statistic. Only three modules vary between the results obtained using the two methods. P-value is illustrated by the intensity of the color and the effect size by the size of the dot. Only the modules with p-value for the enrichment lower than 0.005 are shown. The modules are described by the titles followed by the original number of genes in module and ID. **(B)** The three modules varying in the results obtained by disco.score and t-statistic. The classification of module LI.M4.2 as concordant as well as classification of modules LI.M222 and LI.M87 as discordant was detected by disco.score, but not by t-statistic gene sorting.

A

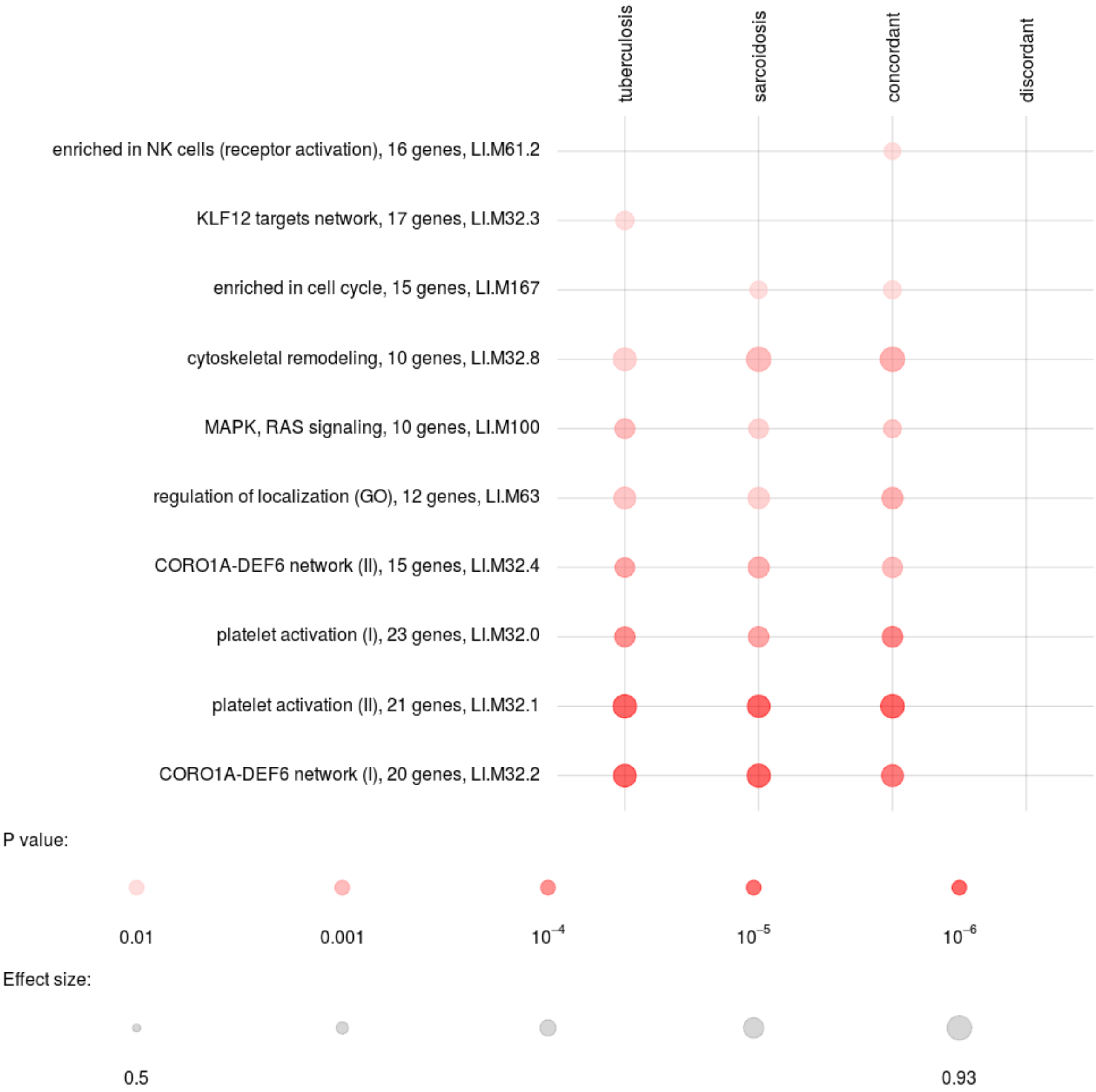
tuberculosis
sarcoidosis
concordant
discordant
enriched in NK cells (receptor activation), 16 genes, LI.M61.2
KLF12 targets network, 17 genes, LI.M32.3
enriched in cell cycle, 15 genes, LI.M167
cytoskeletal remodeling, 10 genes, LI.M32.8
MAPK, RAS signaling, 10 genes, LI.M100
regulation of localization (GO), 12 genes, LI.M63
CORO1A-DEF6 network (II), 15 genes, LI.M32.4
platelet activation (I), 23 genes, LI.M32.0
platelet activation (II), 21 genes, LI.M32.1
CORO1A-DEF6 network (I), 20 genes, LI.M32.2
P value:
0.01
0.001
10^{-4}
10^{-5}
10^{-6}
Effect size:
0.5
0.93

B

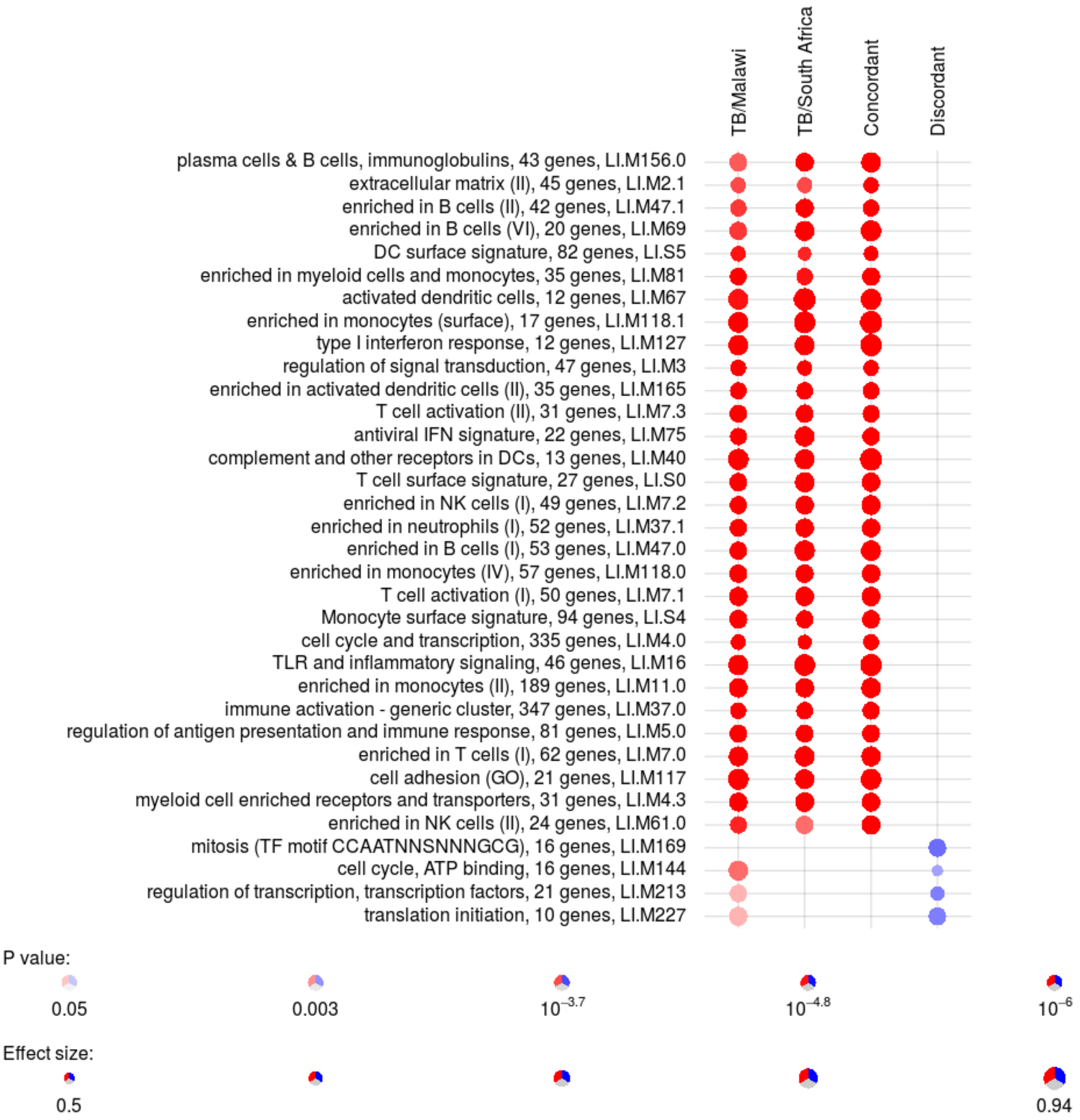

Figure S5 Disco.score-based concordance detection illustrates known biological background of disease similarity

Visualization of modules enriched in the validation tests. P-value is illustrated by the intensity of the color and the effect size by the size of the dot.

(A) Modules enriched in test data sets derived from GEO (Maertzdorf et al., 2012, GSE34608). The gene modules enriched in TB patients, sarcoidosis patients, concordant gene modules identified with disco.score among the two groups of patients and discordant gene modules identified with disco.score are presented in the picture. (**B)** Modules enriched in test data sets derived from GEO (Kaforou et al., 2013; GSE19491). The gene modules enriched in TB patients from Malawi (TB/Malawi), TB patients from South Africa (TB/South Africa), concordant (red) and discordant (blue) gene modules identified with disco.score in the two groups of patients are presented in the picture. The modules are described by the titles followed by the original number of genes in module and ID.

A

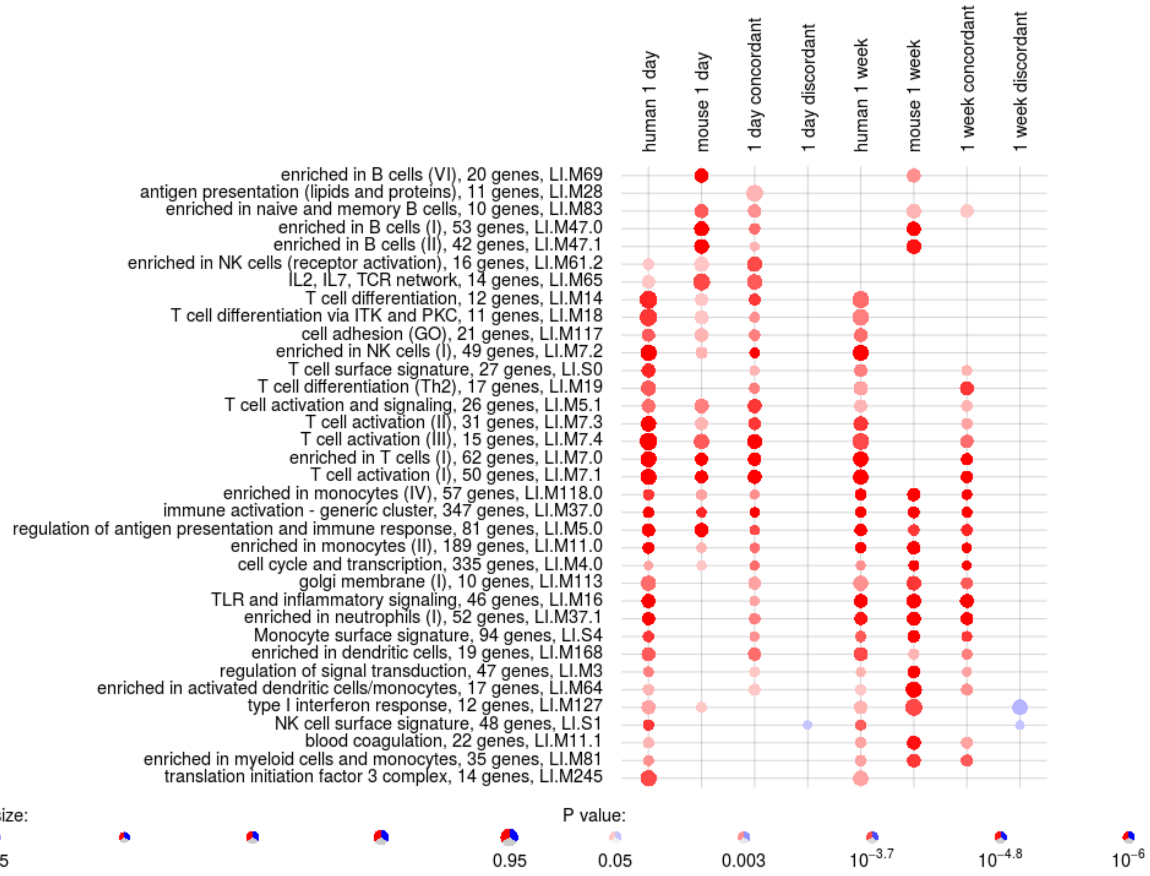
human 1 day
mouse 1 day
1 day concordant
1 day discordant
human 1 week
mouse 1 week
1 week concordant
1 week discordant
enriched in B cells (VI), 20 genes, LI.M69
antigen presentation (lipids and proteins), 11 genes, LI.M28
enriched in naive and memory B cells, 10 genes, LI.M83
enriched in B cells (I), 53 genes, LI.M47.0
enriched in B cells (II), 42 genes, LI.M47.1
enriched in NK cells (receptor activation), 16 genes, LI.M61.2
IL2, IL7, TCR network, 14 genes, LI.M65
T cell differentiation, 12 genes, LI.M14
T cell differentiation via ITK and PKC, 11 genes, LI.M18
cell adhesion (GO), 21 genes, LI.M117
enriched in NK cells (I), 49 genes, LI.M7.2
T cell surface signature, 27 genes, LI.S0
T cell differentiation (Th2), 17 genes, LI.M19
T cell activation and signaling, 26 genes, LI.M5.1
T cell activation (II), 31 genes, LI.M7.3
T cell activation (III), 15 genes, LI.M7.4
enriched in T cells (I), 62 genes, LI.M7.0
T cell activation (I), 50 genes, LI.M7.1
enriched in monocytes (IV), 57 genes, LI.M118.0
immune activation - generic cluster, 347 genes, LI.M37.0
regulation of antigen presentation and immune response, 81 genes, LI.M5.0
enriched in monocytes (II), 189 genes, LI.M11.0
cell cycle and transcription, 335 genes, LI.M4.0
golgi membrane (I), 10 genes, LI.M113
TLR and inflammatory signaling, 46 genes, LI.M16
enriched in neutrophils (I), 52 genes, LI.M37.1
Monocyte surface signature, 94 genes, LI.S4
enriched in dendritic cells, 19 genes, LI.M168
regulation of signal transduction, 47 genes, LI.M3
enriched in activated dendritic cells/monocytes, 17 genes, LI.M64
type I interferon response, 12 genes, LI.M127
NK cell surface signature, 48 genes, LI.S1
blood coagulation, 22 genes, LI.M11.1
enriched in myeloid cells and monocytes, 35 genes, LI.M81
translation initiation factor 3 complex, 14 genes, LI.M245
Effect size:
0.5
0.95
P value:
0.05
0.003
10^-3.7
10^-4.8
10^-6

B

Concordant module, detected by disco.score and by methods of Seok et al. and Takao et al.[4,5]

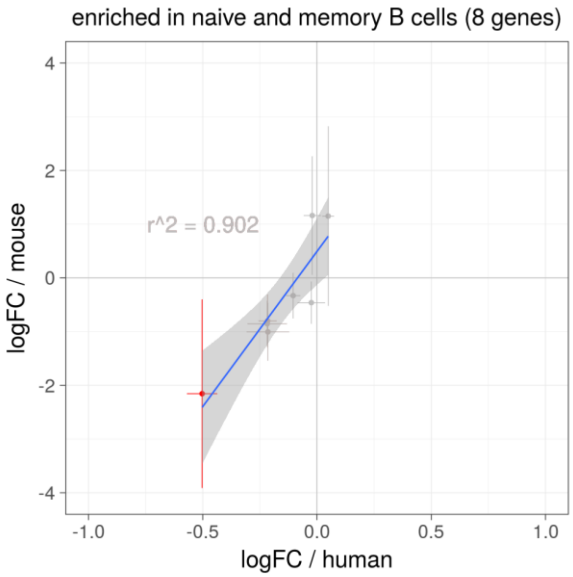

Discordant module, detected by only disco.score

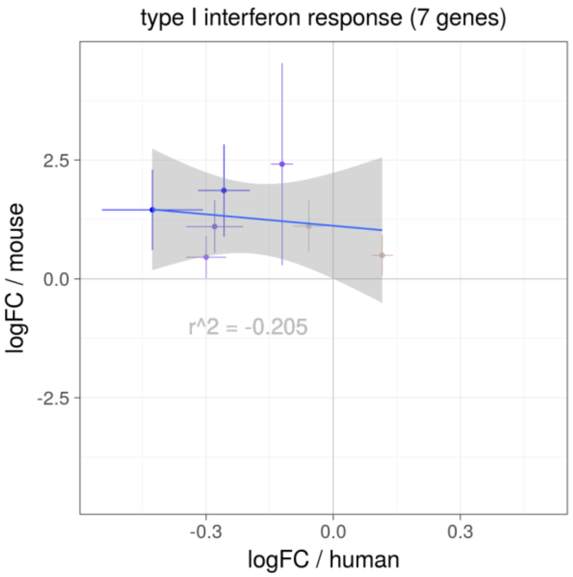

Figure S6 Disco.score-based compared with correlation-based results of data sets comparison

(A) Concordant (red) and discordant (blue) modules enriched in data sets derived from GEO (Calvano et al., 2005; GSE3284). The gene modules enriched in patients after burn and mouse model of burn in time points of 1 day and 1 week are presented in the picture. P-value in illustrated by the intensity of the color and the effect size by the size of the dot. Only the modules with p-value for the enrichment smaller than 10^-7 are shown. The modules are described by the titles followed by the original number of genes in module and ID. **(B)** One week after infection, module “enriched in naïve and memory B cells” was detected as concordant by disco.score and B-cell receptor signaling was described as correlating by Seok et al. and Takao et al.[4,5]. Module “type I interferon response” was detected as discordant by disco.score, which was not detected by the correlation-based methods.

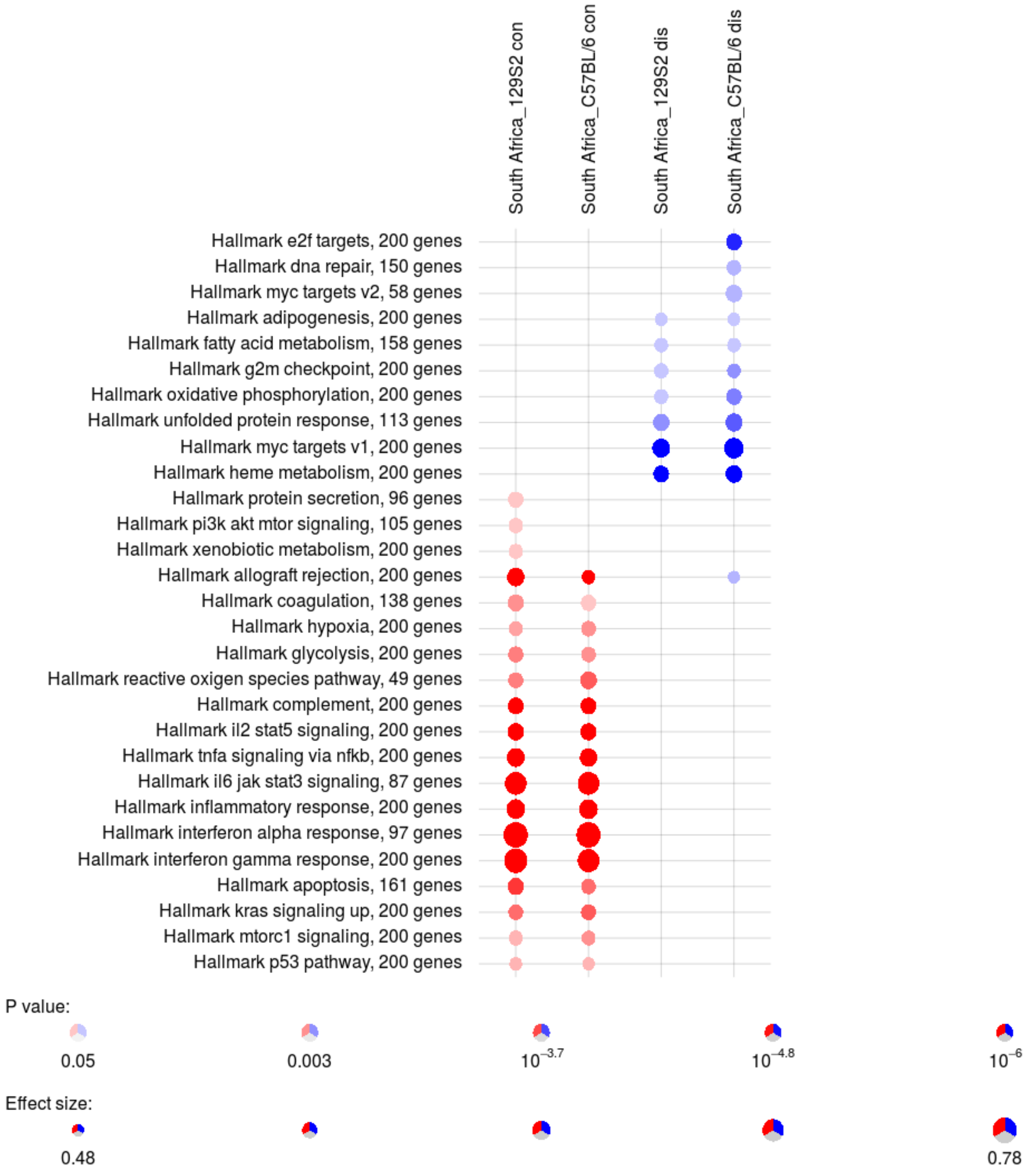

Figure S7 Results of disco.score based module detection with use of MSigDB modules in comparison of human and murine data sets

Concordant (red) and discordant (blue) MSigDB Hallmark Gene Sets enriched in human WB data set from South Africa and WB data sets from C57BL/6 and 129S2 mice at day 21 p.i. The modules “Hallmark IL2 STAT5 signaling” and “Hallmark IL6 STAT3 signaling” are concordant in comparison of South African cohort to both 129S2 and C57BL/6 strains. The modules are described by the titles followed by the original number of genes in module and ID.

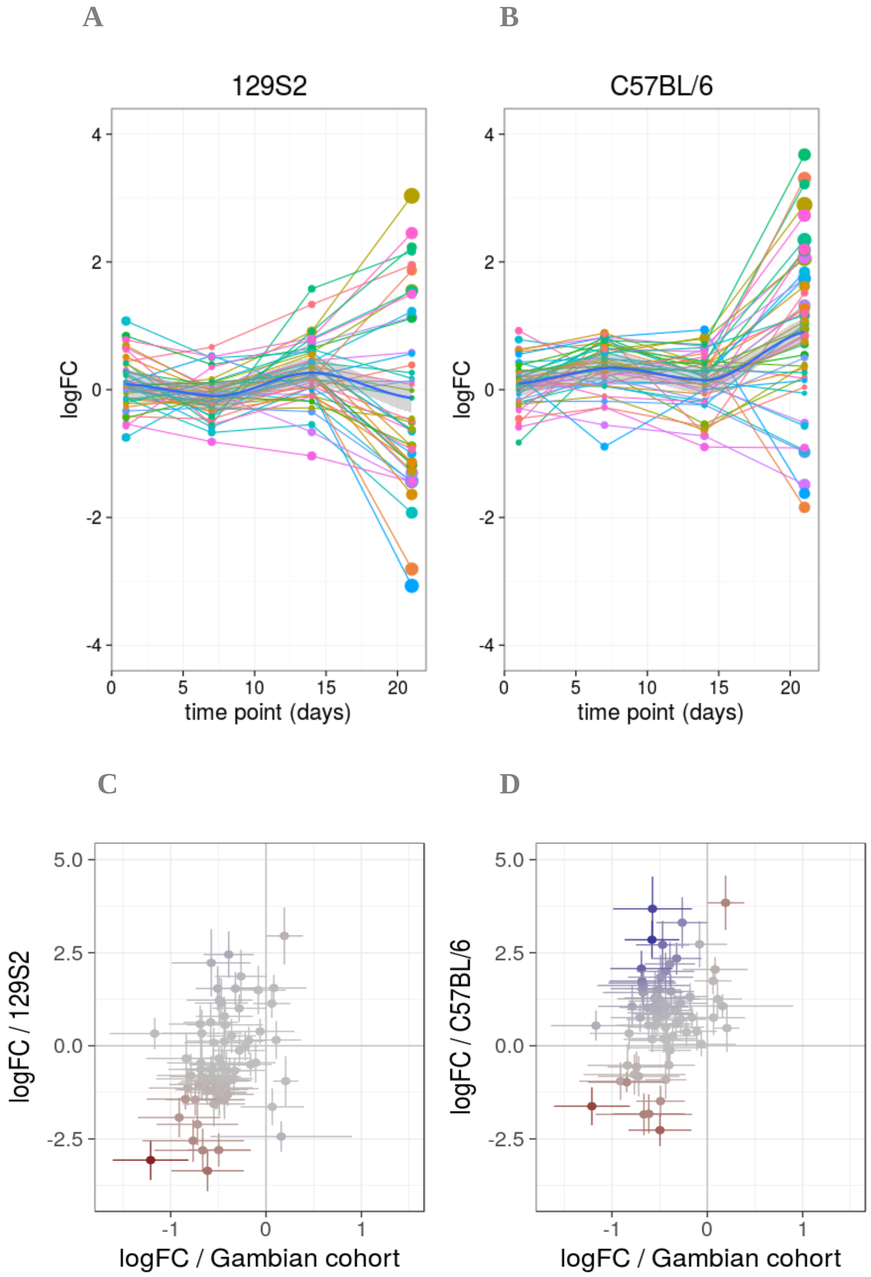

Figure S8 Regulation of many T-cell related genes is concordant between human and 129S2, but discordant between human and C57BL/6 mice

Expression of genes belonging to the modules "enriched in T cells (I)", "T cell activation and signaling" and "T cell activation (I)". **Upper panel:** Gene Expression in the time points day 1, day 7, day 14 and day 21 p.i. in (**A)** susceptible, (**B)** resistant mouse strain. Different colors represent different genes belonging to at least one of the modules. The trend line is shown in blue. **Lower panel:** Log fold changes of gene expression of the (**C)** susceptible and (**D)** resistant mouse plotted again log fold changes of gene expression of the cohort from The Gambia. The intensity of the color represents disco.score. Bars represent 95% CI for the log fold change.

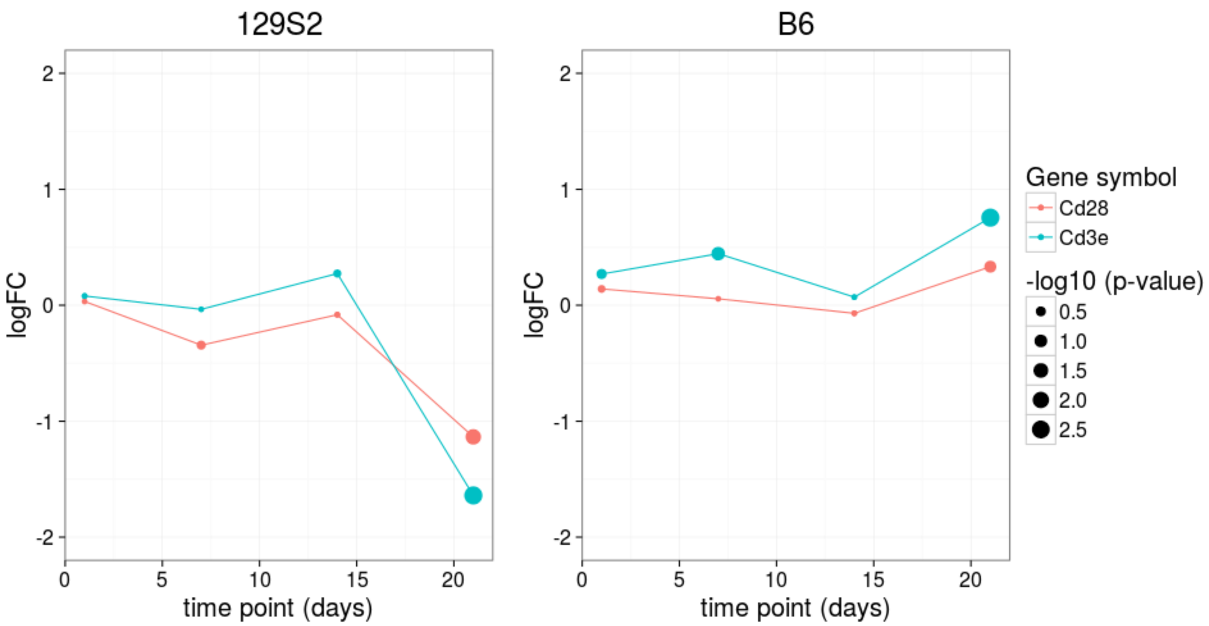

Figure S9 Expression of Cd28 and Cd3e genes in 129S2 and C57BL/6 mice

Expression of the Cd28 and Cd3e genes in the susceptible and resistant mouse strain. The expression regulation of these genes was found as discordant between susceptible and resistant macaque lineages in the study of Javed et al. (2016).

A

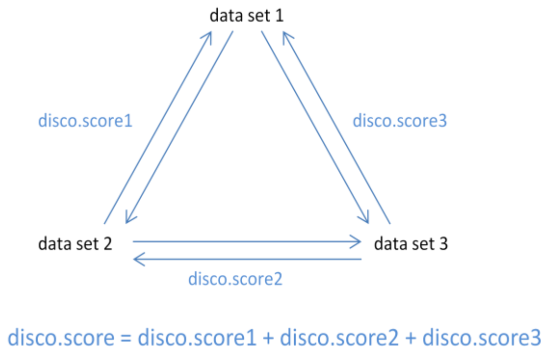

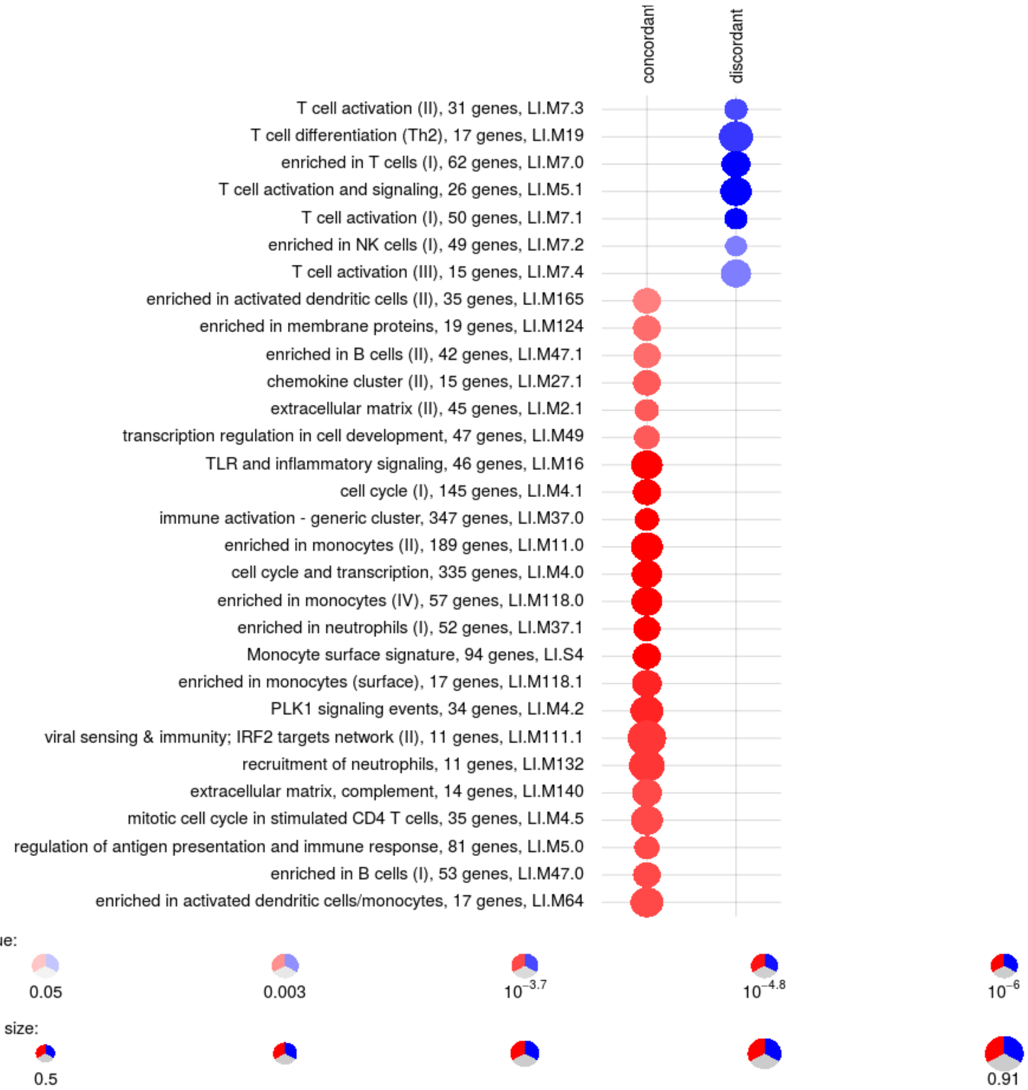

B

Figure S10 Disco.score application for multiple comparisons

(A) Method of calculation of disco.score for three comparisons. **(B)** Results of comparison of three data sets by disco.score followed by GSE. The data sets compared are human WB data from The Gambian cohort, 129S2 mouse strain WB data 21days p.i. and C57BL/6 mouse strain WB data 21 days p.i. The concordant modules among the three data sets are marked red, the discordant modules among the three data sets are marked blue. The results from triple comparison reflect the results of pair-wise comparisons presented in the Results section.

Table S1 Compared data sets and analysis results. r^2 and r values refer to correlation calculated as in studies [4,5]. tp – time point.

Comparison	Human data set	Mouse data set	Human tissue	Mouse tissue	Human tp	Mouse tp	# 1:1 orthologs	r^2	included genes	r	included genes	Concordant modules	Discordant modules
1	Gambia	B6	blood	blood	NA	day 1	14712	0.001	5709	0.05	221	14	47
2	Gambia	129S	blood	blood	NA	day 1	14712	0.003	6368	0.163	699	29	29
3	Malawi	B6	blood	blood	NA	day 1	15004	0.003	4381	0.238	211	20	42
4	Malawi	129S	blood	blood	NA	day 1	15004	0.023	5212	0.343	704	28	36
5	South Africa	B6	blood	blood	NA	day 1	15004	0	5570	0.081	251	19	52
6	South Africa	129S	blood	blood	NA	day 1	15004	0.002	6332	0.067	813	36	28
7	Gambia	B6	blood	blood	NA	day 7	14712	0.024	5658	-0.215	275	14	45
8	Gambia	129S	blood	blood	NA	day 7	14712	0	5798	-0.064	289	53	8
9	Malawi	B6	blood	blood	NA	day 7	15004	0.003	4399	-0.042	228	21	33
10	Malawi	129S	blood	blood	NA	day 7	15004	0.004	4483	0.037	254	49	16
11	South Africa	B6	blood	blood	NA	day 7	15004	0.007	5542	-0.156	314	24	41
12	South Africa	129S	blood	blood	NA	day 7	15004	0	5650	-0.089	316	66	14
13	Gambia	B6	blood	blood	NA	day 14	14712	0.001	6720	0.076	1065	53	35
14	Gambia	129S	blood	blood	NA	day 14	14712	0	7019	0.148	1248	66	28
15	Malawi	B6	blood	blood	NA	day 14	15004	0.002	5764	0.152	1008	56	25
16	Malawi	129S	blood	blood	NA	day 14	15004	0.012	5877	0.268	1066	60	25
17	South Africa	B6	blood	blood	NA	day 14	15004	0	6754	0.081	1247	60	27
18	South Africa	129S	blood	blood	NA	day 14	15004	0.006	6905	0.237	1267	67	24
19	Gambia	B6	blood	blood	NA	day 21	14712	0	10334	0.08	3772	90	27
20	Gambia	129S	blood	blood	NA	day 21	14712	0.009	10409	0.231	3519	106	5
21	Malawi	B6	blood	blood	NA	day 21	15004	0.009	1000	0.218	7979	78	29
22	Malawi	129S	blood	blood	NA	day 21	15004	0.033	9708	0.363	1698	102	2
23	South Africa	B6	blood	blood	NA	day 21	15004	0.004	10535	0.165	3753	74	29
24	South Africa	129S	blood	blood	NA	day 21	15004	0.025	10235	0.331	3400	104	4
25	Gorilla	GSE23508	THP1	BMDM	24h	24h	14314	0.031	10395	0.296	3821	71	17
26	Gorilla	GSE47673	THP1	BMDM	6h	6h	13881	0.042	5779	0.417	488	34	0
27	GSE11199 LTB	GSE23508	MDM	BMDM	4h	24h	11695	0.032	4945	0.314	159	54	4
28	GSE11199 LTB	GSE47673	MDM	BMDM	4h	6h	11630	0.068	4352	0.242	135	39	0
29	GSE11199 PTB	GSE23508	MDM	BMDM	4h	24h	11688	0.052	4972	0.369	214	57	15
30	GSE11199 PTB	GSE47673	MDM	BMDM	4h	6h	11409	0.052	4582	0.374	162	40	2
31	GSE11199 TBM	GSE23508	MDM	BMDM	4h	24h	11694	0.061	4949	0.468	158	66	18
32	GSE11199 TBM	GSE47673	MDM	BMDM	4h	6h	11416	0.081	4542	0.367	126	46	3
33	Gorilla	GSE47673	THP1	BMDM	1h	1h	13885	0	5739	0.559	101	29	0
34	GSE11199 LTB	GSE47673	MDM	BMDM	4h	1h	11417	0.002	375	0.119	24	32	3
35	GSE11199 PTB	GSE47673	MDM	BMDM	4h	1h	11412	0.012	451	0.319	21	37	5
36	GSE11199 TBM	GSE47673	MDM	BMDM	4h	1h	11419	0.021	373	0.177	22	41	7

Table S2 Characteristics of the simulated concordant and discordant modules

Concordance	# of genes	% of regulated genes
concordant	10	10
concordant	10	20
concordant	10	30
concordant	20	10
concordant	20	20
concordant	20	30
concordant	30	10
concordant	30	20
concordant	30	30
concordant	40	10
concordant	40	20
concordant	40	30
concordant	50	10
concordant	50	20
concordant	50	30
discordant	10	10
discordant	10	20
discordant	10	30
discordant	20	10
discordant	20	20
discordant	20	30
discordant	30	10
discordant	30	20
discordant	30	30
discordant	40	10
discordant	40	20
discordant	40	30
discordant	50	10
discordant	50	20
discordant	50	30
